# *Wolffia globosa* Ethanolic Extract Protects Against Bisphenol A-Induced Osteoblast Dysfunction via Antioxidant Defense, Apoptosis Inhibition, and β-Catenin Modulation

**DOI:** 10.3390/ijms27125352

**Published:** 2026-06-13

**Authors:** Benjawan Wudtiwai, Pornsiri Pitchakarn, Piya Temviriyanukul, Pattaralawan Sittiju, Woorawee Inthachat, Jirarat Karinchai, Nuttida Phunsanit, Prachya Kongtawelert, Peraphan Pothacharoen

**Affiliations:** 1 Center of Multidisciplinary Technology for Advanced Medicine (CMUTEAM), Faculty of Medicine, Chiang Mai University, Chiang Mai 50200, Thailand; benjawan.w@cmu.ac.th; 2 Department of Biochemistry, Faculty of Medicine, Chiang Mai University, Muang Chiang Mai, Chiang Mai 50200, Thailand; pornsiri.p@cmu.ac.th (P.P.); pattaralawan.s@cmu.ac.th (P.S.); jirarat.k@iesa.ac.th (J.K.); nuttida.ph@cmu.ac.th (N.P.); prachya.k@cmu.ac.th (P.K.); 3 Institute of Nutrition, Mahidol University, Salaya, Nakhon Pathom 73170, Thailand; piya.tem@mahidol.ac.th (P.T.); woorawee.int@mahidol.ac.th (W.I.); 4 Division of Clinical Chemistry, Department of Medical Technology, Faculty of Associated Medical Sciences, Chiang Mai University, Chiang Mai 50200, Thailand; 5 Faculty of Pharmacy, Institute of Entrepreneurial Science Ayutthaya, Phra Nakhon Si Ayutthaya 13000, Thailand

**Keywords:** endocrine-disrupting chemicals, oxidative stress, osteogenesis, osteotoxicity, Wnt/β-catenin pathway

## Abstract

The prevalent endocrine disruptor bisphenol A (BPA) is associated with aging-related conditions, including metabolic disorders. It has been shown that BPA promotes bone fragility through oxidative stress-induced apoptosis and impaired osteoblast differentiation. The identification of sustainable bioactive substances that alleviate BPA-induced bone toxicity is thus of biomedical and environmental significance. *Wolffia globosa* (WG), the world’s smallest flowering aquatic plant, has recently gained attention as a high-protein, antioxidant-rich nutraceutical, yet its impact on BPA-induced osteoblast dysfunction has not been systematically investigated. This study presents a comprehensive assessment of WG ethanolic extract (WGE) in MC3T3-E1 pre-osteoblasts, incorporating thorough phytochemical characterization, acute high-dose and chronic low-dose BPA exposure models, and multi-faceted mechanistic analysis. LC-MS/MS profiling identified luteolin (116.17 ± 0.69 µg/g), rosmarinic acid (54.80 ± 2.12 µg/g), and apigenin (48.77 ± 0.61 µg/g) as the predominant bioactive compounds. WGE exhibited potent antioxidant capacity across DPPH and ABTS radical scavenging assays, complemented by high ORAC and FRAP values, reflecting broad-spectrum antioxidant mechanisms. Treatment with WGE (25 and 50 µg/mL) resulted in significant alleviation of BPA-induced cytotoxicity, decreased intracellular ROS levels, and inhibited apoptosis. WGE (12.5 µg/mL) also modulated autophagy-related markers (LC3-II, Beclin-1, and p62), suggesting potential autophagic participation, although flux verification was not conducted. Treatment with WGE (12.5 µg/mL) also restored BPA-suppressed osteogenesis under chronic exposure, as evidenced by enhanced alkaline phosphatase activity, and increased both mineralization and upregulation of osteogenic genes including runt-related transcription factor2 (*Runx2*), collagen type I alpha 1 (*Colla1*), alkaline phosphatase (*ALP*), and osteocalcin (*OCN*). These effects were accompanied by partial reactivation of Wnt/β-catenin signaling. This study is the first to demonstrate that WGE protects osteoblasts from BPA toxicity by concurrently strengthening antioxidant defenses, limiting apoptosis, modulating autophagy-related markers, and supporting β-catenin-mediated osteogenesis, highlighting WG as a promising sustainable nutraceutical candidate for the prevention of environmental toxin-related bone fragility.

## 1. Introduction

Osteoporosis and related bone fragility disorders constitute a major public health burden worldwide, particularly in aging societies. Fractures are strongly linked to a reduction in bone strength, which depends on the dynamic balance between bone formation and resorption [1]. Osteoblasts are central to matrix production and mineralization, and their lineage commitment and differentiation are regulated by transcription factors such as Runx2 and Sp7. Any disruption to their viability or maturation is a critical impairment to skeletal fragility [2]. Osteoblast dysfunction is therefore a key driver of bone fragility and is highly susceptible to both aging-related and environmental stressors.

While age and hormonal changes are well-established contributors to osteoporosis, emerging evidence indicates that endocrine-disrupting chemicals (EDCs), for example, bisphenol A (BPA), are insidious environmental stressors of bone health. BPA, a ubiquitous plasticizer frequently detected in human fluids and tissues, has been increasingly linked epidemiologically and experimentally to reduced bone mineral density and impaired osteoblast activity, suggesting that environmental toxicants may compound age-related bone deterioration [3,4,5,6].

There is increasing evidence to indicate that BPA not only interferes with osteoblast function but also acts as an environmental accelerator of cellular aging. BPA causes oxidative stress, which leads to cellular senescence characterized by increased expression of p16, p21, and cyclin-dependent kinase inhibitors (CDK inhibitors), which regulate cell cycle arrest and cellular senescence, as well as the activation of unfolded protein response pathways (PERK-ATF4-CHOP). These changes, which promote premature aging phenotypes, are implicated in metabolic and cardiovascular disease [7,8]. Experimental and epidemiological studies have demonstrated that BPA and related compounds contribute to telomere shortening, impaired DNA repair, mitochondrial dysfunction, and immune dysregulation, hallmarks of accelerated aging that increase susceptibility to inflammation, metabolic disorders, and degenerative diseases [9,10,11]. Collectively, these aging-related stress programs may compromise the long-term regenerative capacity of osteoblasts.

At a cellular level, exposure to BPA precipitates mitochondrial dysfunction, excessive accumulation of ROS, and initiation of apoptosis cascades, including those impacting caspase-3 activation and PARP cleavage, events that reduce the survival capacity of osteoblasts [12,13]. BPA also perturbs autophagy, a quality-control pathway essential for the turnover of damaged proteins and organelles and osteoblast differentiation [14]. These stress responses interface with and ultimately suppress the Wnt/β-catenin pathway, an indispensable pathway for osteoblast differentiation and matrix mineralization [15]. Inhibition of β-catenin consequently diminishes downstream osteogenic gene programs, including *Runx2*, *Col1a1*, *ALP*, and *OCN*, thereby compromising bone-forming capacity [16].

Given the multifactorial nature of BPA-induced toxicity, there has been an increasing interest in natural bioactive compounds and their capacity to counter oxidative, apoptotic, and differentiation-related stress [17,18]. *Wolffia globosa* (Kai-pum in Thai; WG), an antioxidant-rich edible microplant, contains phenolics, flavonoids, proteins, and micronutrients with well-documented redox-modulating and anti-inflammatory and metabolic regulatory properties [19,20]. These phytochemical constituents contribute to a decrease in intracellular ROS, the enhancement of antioxidant enzymes such as HO-1 and SOD-1, suppression of cytokine-driven inflammatory signaling (e.g., NF-κB), stabilization of mitochondrial function, and reinforcement of osteogenic pathways [17,21,22]. Among its constituents, the flavones luteolin and apigenin and the phenolic acid rosmarinic acid have been reported to scavenge ROS, enhance antioxidant defenses, and suppress pro-inflammatory signaling, including NF-κB and MAPK pathways, while also promoting osteoblast differentiation and mineralization and inhibiting osteoclastogenesis in various bone models [23]. Evidence pertinent to the impact of natural products with similar polyphenolic and flavonoid profiles demonstrates substantial protection against BPA-induced oxidative damage, apoptosis, mitochondrial dysfunction, and autophagy disruption across multiple cell types [24,25]. In this context, the antioxidant-rich profile of *Wolffia globosa* positions WGE as a promising sustainable nutraceutical capable of attenuating BPA-induced osteoblast dysfunction by providing antioxidant defense, anti-apoptotic activity, modulation of autophagy, and restoration of Wnt/β-catenin-driven osteogenic signaling [26].

Despite increasing evidence, comprehensive investigation into WGE’s multi-targeted modulation of oxidative stress, apoptosis, autophagy, and osteogenic signaling under BPA exposure is limited. More experimental evidence is vital to the understanding of how sustainable nutraceuticals can counteract the multifactorial osteotoxic and aging-accelerating effects of BPA.

Given these gaps, there is a need for sustainable nutraceutical candidates that can counteract BPA-induced osteoblast dysfunction through coordinated actions on oxidative stress, cell death, autophagy, and osteogenic signaling. *Wolffia globosa*, an edible microplant with a high protein and phenolic content, has emerged as an antioxidant-rich food source; however, its effects on BPA-induced osteotoxicity and the underlying mechanisms have not been comprehensively defined. We hypothesized that WGE mitigates BPA-induced osteoblast dysfunction by (i) strengthening antioxidant defenses, (ii) attenuating apoptosis, (iii) modulating autophagy-related markers, and (iv) reactivating Wnt/β-catenin signaling to restore osteogenic programs. To address this, we combined the detailed phytochemical profiling of WGE with two complementary in vitro BPA exposure models—acute high-dose (50 µM, 12–24 h) and chronic low-dose (10 µM, 7–21 days)—in MC3T3-E1 pre-osteoblasts and evaluated a broad panel of endpoints, including ROS, HO-1/SOD-1, apoptosis, autophagy-related markers, and osteogenic outputs (ALP activity, mineralization, and osteogenic gene expression). This integrated design allows us, for the first time, to define the multi-target protective actions of WGE against BPA-induced osteoblast injury and to position *Wolffia globosa* as a promising sustainable nutraceutical for the mitigation of environmental toxin-related bone fragility.

## 2. Results

### 2.1. Phytochemical Characterization and Antioxidant Properties of WGE

Ethanolic extraction of *Wolffia globosa* (WG) yielded 18.75% (*w*/*w*) dry extract, indicating efficient recovery of bioactive constituents. Phytochemical analyses revealed that WG ethanolic extract (WGE) contained high levels of total phenolic compounds (42.82 ± 3.56 mg gallic acid equivalents (GAE)/g extract) and total flavonoids (17.79 ± 1.96 mg catechin equivalents (CE)/g extract) (Table 1). These values are comparable to those reported from polyphenol-rich aquatic and terrestrial plants with established antioxidant potential [27], demonstrating potent antioxidant capabilities, suggesting that WG is a potential substantial source of antioxidant-rich metabolites.

The phytochemical profiles of WGE were studied using LC-MS/MS techniques. Rosmarinic acid, luteolin, and apigenin were detected in WGE at 54.80 ± 2.12, 116.17 ± 0.69, and 48.77 ± 0.61 µg/g extract, respectively (Table 2). Luteolin was the predominant compound in the extract, followed by rosmarinic acid and apigenin, which were present in comparable quantities.

In free-radical scavenging assays, WGE demonstrated notable antioxidant activity, evidenced by SC_50_ values of 40.47 ± 7.50 µg/mL for ABTS and 247.27 ± 33.81 µg/mL for DPPH (Table 3). The significantly lower SC_50_ in the ABTS assay suggests superior scavenging efficacy in aqueous conditions, consistent with the rich hydrophilic phenolic content of the extract. The use of multiple assays, DPPH, ABTS, ORAC, and FRAP, provides a comprehensive evaluation of antioxidant capacity, as each method probes different antioxidant mechanisms and radical types. DPPH and ABTS assays are based on electron transfer and measure the ability to quench stable free radicals, with ABTS being more applicable in aqueous environments, highlighting water-soluble antioxidants. ORAC assesses antioxidant capacity via hydrogen atom transfer against biologically relevant peroxyl radicals, reflecting in vivo antioxidant potential. FRAP measures reducing power by electron donation, indicating the potential to reduce ferric ions. The high ORAC value (1725.85 ± 99.76 µmol Trolox/g extract) and FRAP value (210.07 ± 2.62 µmol Trolox/g extract) further highlight the robust antioxidant capacity of WGE through multiple pathways.

Collectively, these findings underscore that WGE is a phenolic- and flavonoid-rich extract possessing broad-spectrum and potent radical scavenging properties. This biochemical profile supports its potential protective role against oxidative stress in osteogenic regulation, suggesting it warrants further investigation and its incorporation into subsequent cell-based assays.

### 2.2. WGE Induces the Expression of HO-1 and SOD-1 in MC3T3-E1 Osteoblasts

**Figure 1:**
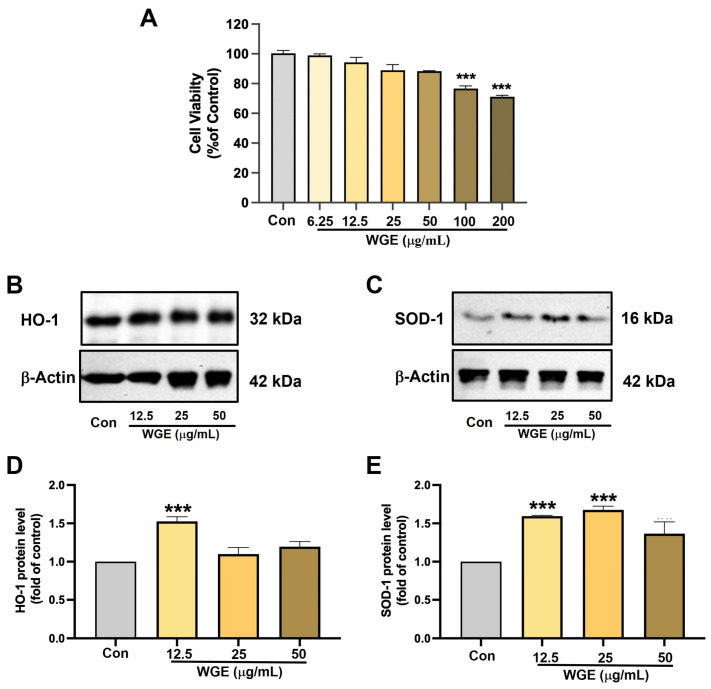
The effect of WGE on (**A**) cell viability and the expression level of key antioxidant enzymes, (**B**,**D**) HO-1 and (**C**,**E**) SOD-1 of MC3T3-E1 osteoblasts. (**A**) Cell viability assessed by MTT assay following 24 h of treatment with WGE at concentrations ranging from 6.25 to 200 µg/mL to determine non-cytotoxic doses. (**B**,**C**) Representative Western blots and (**D**,**E**) densitometric quantification of the level of expression of HO-1 (32 kDa) and SOD-1 (16 kDa), respectively, in cells treated with 12.5 to 50 µg/mL WGE for 24 h. β-Actin (42 kDa) was used as a loading control, with molecular weight markers indicated. Protein levels were normalized to β-actin and are presented as fold change relative to the control group (0.1% DMSO). Data are represented as mean ± SD from three independent experiments (n = 3). Statistical significance was determined by one-way ANOVA followed by Tukey’s post hoc test. *** *p* < 0.001 vs. control.

WGE (6.25–50 µg/mL, 24 h) did not significantly reduce MC3T3-E1 cell viability, which remained at 88–100% of control, whereas 100 and 200 µg/mL caused a modest but significant decrease (24% and 29%, respectively; *p* < 0.001; Figure 1A). WGE was therefore used at ≤50 µg/mL in subsequent experiments. Western blot analysis showed that WGE increased the levels of HO-1 protein in a concentration-dependent manner, with significant induction at non-cytotoxic doses (Figure 1B–D). The level of expression of SOD-1 was also significantly upregulated at 12.5 and 25 µg/mL WGE (*p* < 0.001; Figure 1C–E).

### 2.3. Protective Effects of WGE Against BPA-Induced Cytotoxicity in MC3T3-E1 Osteoblasts

**Figure 2:**
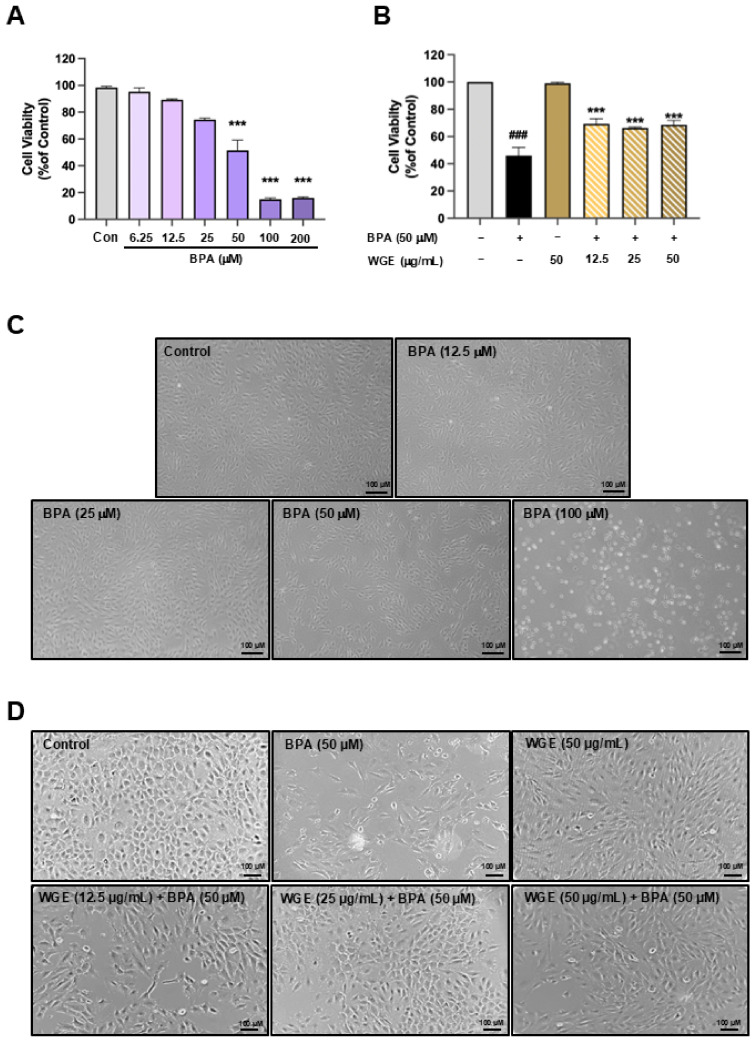
Cytoprotective effects of WGE against BPA-induced cytotoxicity in MC3T-E1 osteoblasts. (**A**) Dose-dependent decrease in cell viability following BPA exposure (6.25–200 µM, 24 h); IC_50_ ≈ 45 µM. (**B**) Pretreatment with WGE (12.5–50 µg/mL, 2 h) significantly restored cell viability after BPA (50 µM, 24 h) exposure. (**C**,**D**) Representative phase-contrast microscope images showing BPA-induced cellular shrinkage and detachment, whereas WGE co-treatment preserved normal cell morphology and confluence. Scale bar = 100 µm. Data represented as mean ± SD from three independent experiments (n = 3). Statistical significance was determined by one-way ANOVA followed by Tukey’s post hoc test. ^###^ *p* < 0.001 vs. control; *** *p* < 0.001 vs. BPA alone.

The chosen concentration range (6.25–200 µM) and the use of 50 µM as a mechanistic stress dose are consistent with previous BPA studies in bone and other tissues (Table S1). MC3T3-E1 cells exposed to BPA (6.25–200 µM, 24 h) exhibited a significant, dose-dependent decrease in viability, with an estimated IC_50_ of ~45 µM; 50 µM BPA reduced viability to ~51.6% and induced cell shrinkage, rounding, and reduced confluence (Figure 2A,C). This condition (50 µM, 24 h) was therefore used as the standard toxicity model. Pretreatment with WGE (12.5–50 µg/mL, 2 h) significantly increased cell viability after exposure to 69–71% BPA (*p* < 0.001 vs. BPA alone) and preserved osteoblastic morphology and monolayer integrity (Figure 2C,D). Protection index values were 1.53 ± 0.27, 1.46 ± 0.17, and 1.51 ± 0.16 for 12.5, 25, and 50 µg/mL WGE, respectively, indicating a ~1.4–1.5-fold increase in viability compared with BPA alone, without clear dose dependence.

### 2.4. WGE Attenuates BPA-Induced Apoptosis in MC3T3-E1 Osteoblasts

**Figure 3:**
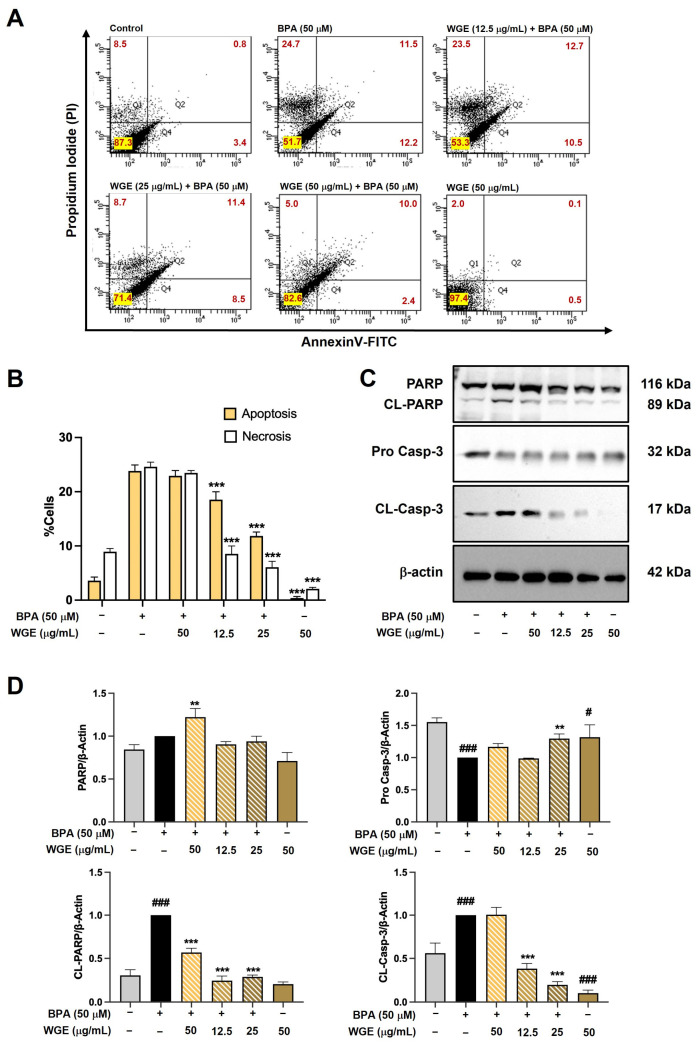
WGE attenuates BPA-induced cell death in MC3T3-E1 osteoblasts. (**A**) Representative Annexin V-FITC/PI flow cytometry dot plots showing distribution of apoptotic cells. BPA treatment (50 µM, 24 h) increased both early and late apoptosis in comparison to controls, while WGE pretreatment (12.5–50 µg/mL) significantly reduced apoptosis in a dose-dependent manner. (**B**) Quantification of total apoptotic cells (early + late apoptosis) expressed as a percentage of the population. (**C**) Western blot analysis of apoptosis-related proteins. BPA induced cleavage of PARP (89 kDa) and caspase-3 (17 kDa) with a concomitant decrease in procaspase-3 (32 kDa). WGE pretreatment inhibited cleavage of PARP and caspase-3 and restored procaspase-3 levels. (**D**) Densitometric quantification of Western blot bands normalized to β-actin and expressed as fold change relative to BPA (for cleaved PARP and cleaved caspase-3) or control (for procaspase-3). Data represented as mean ± SD from three independent experiments (n = 3). Statistical significance was determined by one-way ANOVA followed by Tukey’s post hoc test. ^#^ *p* < 0.05, ^###^ *p* < 0.001 vs. control; ** *p* < 0.01, *** *p* < 0.001 vs. BPA alone.

MC3T3-E1 cells exposed to BPA (50 µM, 24 h) showed a marked increase in total apoptotic cell number compared with controls (23.8% vs. 3.3%), along with a higher PI-positive (necrotic/late apoptotic) population (24.7%) (Figure 3A,B). Co-treatment with WGE (12.5–50 µg/mL) significantly and concentration-dependently reduced total apoptosis to approximately 18.5% at 25 µg/mL and 11.8% at 50 µg/mL and decreased the number of PI-positive cells to about 8.5% and 6.0%, respectively (Figure 3B). Western blot analysis showed that BPA increased the cleaved forms of PARP and caspase-3 while decreasing procaspase-3, whereas WGE pretreatment reduced PARP and caspase-3 cleavage and restored procaspase-3 levels in a concentration-responsive manner (Figure 3C,D).

### 2.5. WGE Suppresses BPA-Induced ROS Accumulation in MC3T3-E1 Osteoblasts

**Figure 4:**
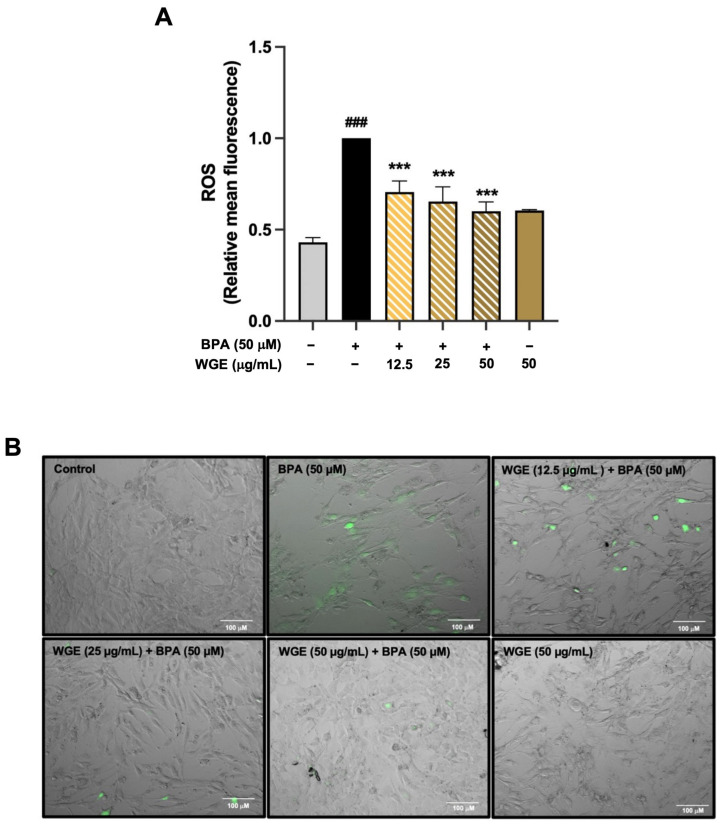
WGE suppresses BPA-induced accumulation of intracellular reactive oxygen species (ROS) in MC3T3-E1 osteoblasts. (**A**) Representative fluorescence micrographs of H_2_DCFDA-stained cells showing intracellular ROS levels. BPA treatment (50 µM, 12 h) markedly increased green fluorescence intensity compared to controls, whereas co-treatment with WGE (12.5, 25, 50 µg/mL) reduced ROS signals in a concentration-dependent manner, restoring fluorescence close to baseline. Scale bar = 100 µm. (**B**) Quantitative analysis of mean fluorescence intensity (MFI) of H_2_DCFDA fluorescence (green) normalized to BPA (set = 1). BPA exposure caused a significant elevation of ROS by approximately 2.3-fold relative to control, while WGE at 25 and 50 µg/mL significantly decreased ROS levels to near basal values. Data represented as mean ± SD from three independent experiments (n = 3). Statistical significance was determined by one-way ANOVA followed by Tukey’s post hoc test. ^###^ *p* < 0.001 vs. control; *** *p* < 0.001 vs. BPA group.

Intracellular ROS levels, assessed by H_2_DCFDA fluorescence, were significantly increased in MC3T3-E1 cells treated with BPA (50 µM, 12 h) in comparison with controls (approximately 2.3-fold; *p* < 0.01; Figure 4A,B). Co-treatment with WGE (12.5–50 µg/mL) reduced ROS in a concentration-dependent manner, with 25 and 50 µg/mL restoring fluorescence intensity close to control levels (*p* < 0.001 vs. BPA alone). WGE at 12.5, 25, and 50 µg/mL decreased ROS intensity by ~1.6-, 1.5-, and 1.4-fold, respectively, relative to BPA-treated cells (Figure 4B).

### 2.6. WGE Suppresses BPA-Disrupted Autophagy and Stress Signaling in MC3T3-E1 Osteoblasts

**Figure 5:**
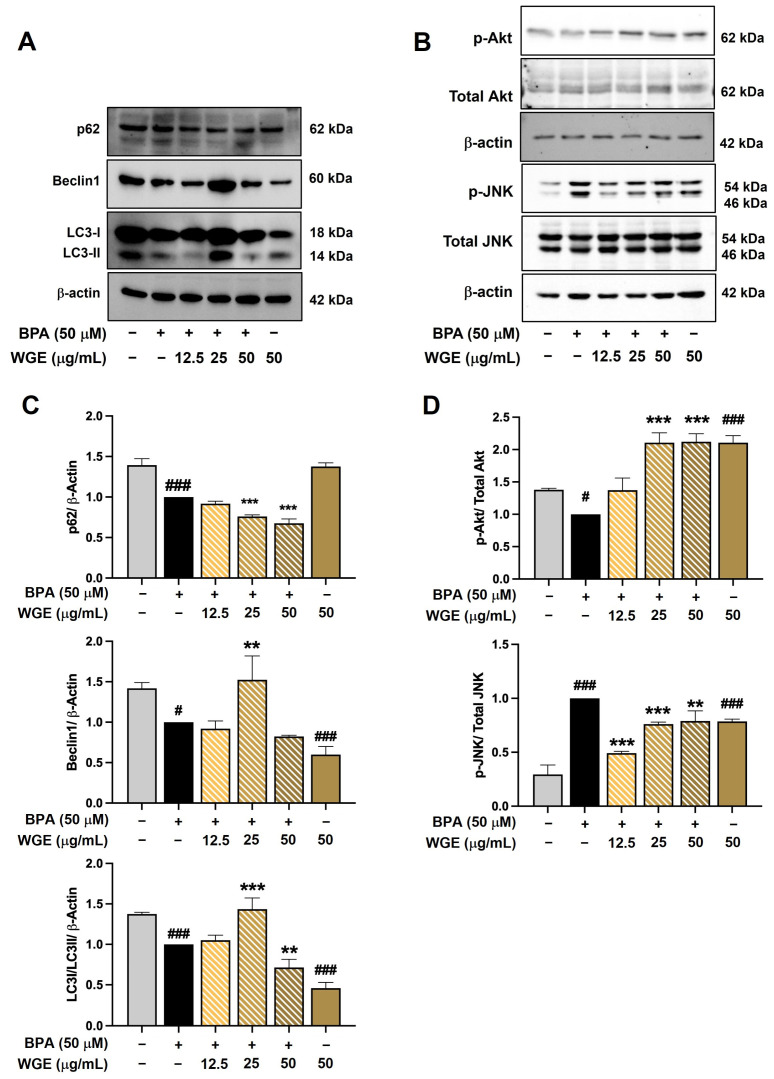
WGE restores autophagy markers and maintains pAkt/pJNK signaling balance in BPA-exposed MC3T3-E1 osteoblasts. (**A**) The expression of p62, Beclin-1, LC3-I/II, and β-actin was measured after the cells were pretreated with WGE (12.5, 25, 50 µg/mL) followed by BPA (50 µM, 12 h) with or without treatment with WGE (12.5, 25, 50 µg/mL). (**B**) Phosphorylated Akt (pAkt, Ser473), total Akt, phosphorylated JNK (pJNK), total JNK, and β-actin under the same conditions. (**C**) Densitometric analysis reveals BPA-induced decreases in LC3-II/LC3-I and Beclin-1 and an increase in p62, all of which were dose-dependently reversed by WGE. (**D**) BPA suppressed pAkt and increased p-JNK levels; these alterations were normalized toward baseline by WGE treatment. Data are presented as mean ± SD of three independent experiments (n = 3). Statistical significance was assessed via one-way ANOVA with Tukey’s post hoc test. ^#^ *p* < 0.05, ^###^ *p* < 0.001 vs. control; ** *p* < 0.01, *** *p* < 0.001 vs. BPA.

MC3T3-E1 cells exposed to BPA (50 µM, 12 h) showed increased p62, reduced Beclin-1, and a lower LC3-II/LC3-I ratio in comparison with controls, together with a decrease in pAkt/Akt and an increased pJNK/JNK (Figure 5A–D). Pretreatment with WGE for 2 h before BPA exposure reduced p62 levels (25–50 µg/mL), increased Beclin-1 and the LC3-II/LC3-I ratio (both significant at 25 µg/mL), and partially restored pAkt/Akt while attenuating pJNK/JNK phosphorylation at all concentrations across 12.5–50 µg/mL (Figure 5A–D). WGE at 50 µg/mL alone, as well as in combination with BPA, increased pAkt/Akt phosphorylation above BPA-inhibited levels without affecting cell viability or apoptosis, indicating that these signaling changes were not associated with additional cytotoxicity (Figure 1, Figure 2 and Figure 3).

### 2.7. WGE Improves Osteogenic Differentiation in BPA-Treated MC3T3-E1 Cells

**Figure 6:**
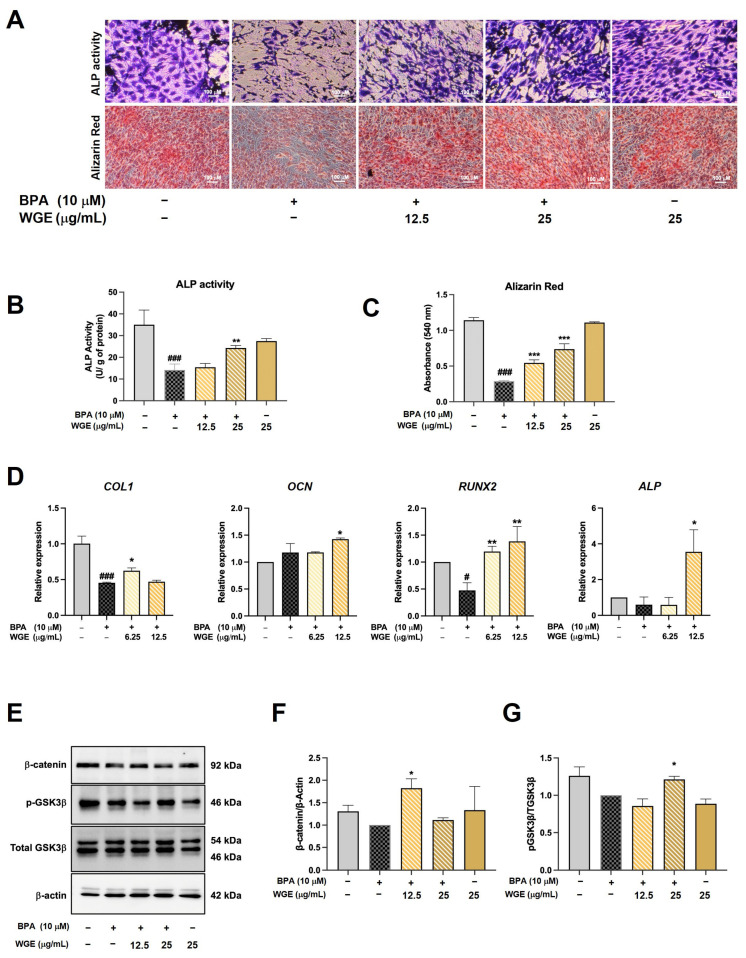
WGE facilitates restoration of osteogenic differentiation inhibited by BPA in MC3T3-E1 osteoblasts. (**A**) Representative images of alkaline phosphatase (ALP) visualized by purple-blue staining (BCIP/NBT substrate) and Alizarin Red S staining showing calcium deposition and mineralized matrix formation after 21 days of osteogenic induction. BPA exposure (10 µM) significantly reduced ALP activity and mineralized nodule formation, whereas WGE treatment (12.5, 25 µg/mL) dose-dependently restored these osteogenic markers. (**B**,**C**) Quantitative analyses of ALP enzymatic activity and Alizarin Red S staining intensity, normalized to control. (**D**) Quantitative real-time PCR analysis of the levels of expression of the mRNA of osteogenic markers *Runx2*, *Col1a1*, *ALP*, and osteocalcin (*OCN*). (**E**) Western blot analysis of β-catenin, total and phosphorylated glycogen synthase kinase 3β (GSK3β), and β-actin. (**F**,**G**) Densitometric quantification of β-catenin protein levels and phosphorylated GSK3β to total GSK3β ratio, normalized to control. BPA reduced β-catenin and p-GSK3β levels, while WGE at 25 µg/mL restored their levels of expression toward basal levels. Data represented as mean ± SD from three independent experiments (n = 3). Statistical significance was determined by one-way ANOVA followed by Tukey’s post hoc test. ^#^ *p* < 0.05, ^###^ *p* < 0.001 vs. control; * *p* < 0.05, ** *p* < 0.01, *** *p* < 0.001 vs. BPA.

MC3T3-E1 pre-osteoblasts were allowed to differentiate for 21 days in osteogenic medium with BPA (10 µM) in the presence or absence of WGE (12.5 or 25 µg/mL). BPA significantly reduced ALP staining and Alizarin Red S-positive mineralized nodules in comparison with controls (*p* < 0.001; Figure 6A–C). Co-treatment with WGE partially restored ALP staining and calcium nodule formation in a concentration-dependent manner, with 25 µg/mL WGE yielding mineralization levels approaching those of untreated cells (*p* < 0.01 and *p* < 0.001 vs. BPA; Figure 6A–C).

At the mRNA level, BPA markedly decreased expression of *Runx2*, *Col1a1*, and *ALP* (*p* < 0.05–0.001 vs. controls), whereas co-treatment with WGE (12.5–25 µg/mL) significantly increased these transcripts relative to BPA alone (*p* < 0.05–0.01; Figure 6D). *OCN* mRNA was significantly elevated in the 25 µg/mL WGE group, consistent with enhanced later-stage differentiation under these conditions (Figure 6D). Western blot analysis showed that BPA (10 µM) modestly reduced β-catenin protein levels and the p-GSK3β/GSK3β ratio; WGE treatment led to a partial increase in β-catenin (significant at 12.5 µg/mL) and p-GSK3β/GSK3β (significant at 25 µg/mL) in comparison with BPA alone (Figure 6E–G).

### 2.8. Molecular Docking

**Figure 7:**
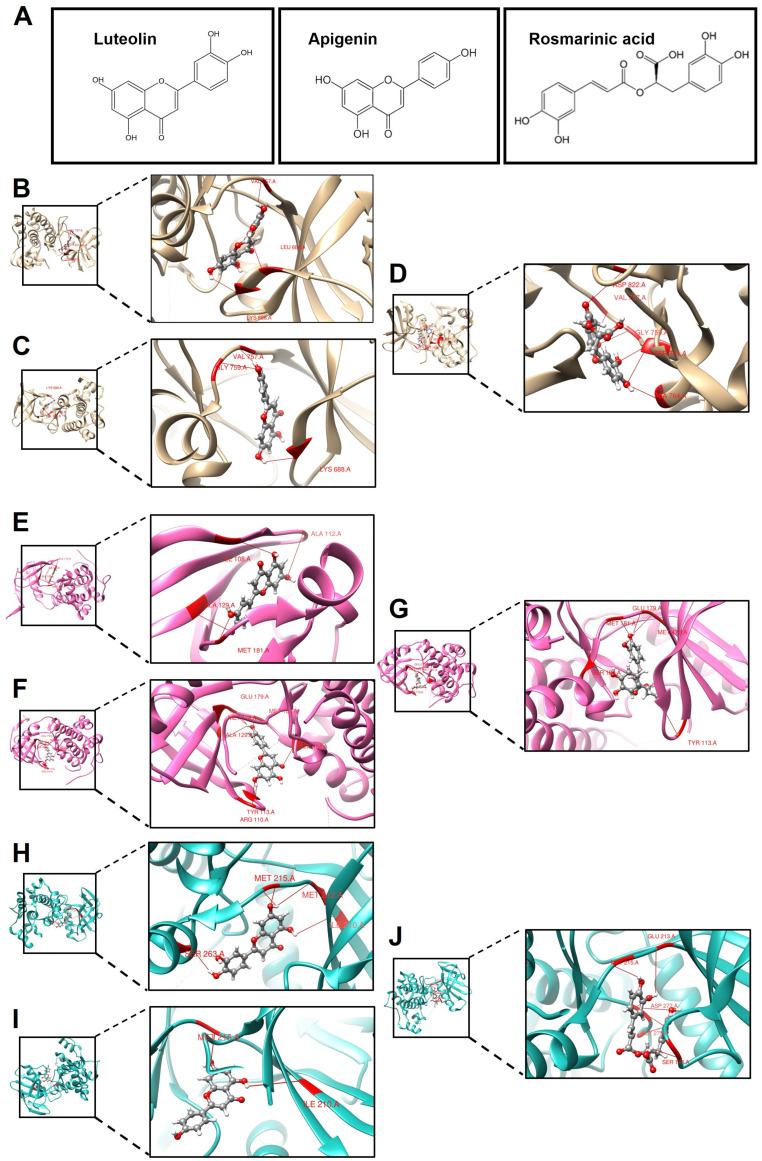
The chemical structure of luteolin, apigenin, and rosmarinic acid (**A**). The 3D structural prediction of the human ASK1 active site in complexes with luteolin (**B**), apigenin (**C**), and rosmarinic acid (**D**). The 3D structural prediction of the human MKK4 active site in complexes with luteolin (**E**), apigenin (**F**), and rosmarinic acid (**G**), and the 3D structural prediction of the human MKK7 active site in complexes with luteolin (**H**), apigenin (**I**), and rosmarinic acid (**J**).

To explore potential molecular mechanisms by which WGE constituents might influence the JNK pathway, molecular docking analyses of luteolin, apigenin, and rosmarinic acid were performed against ASK1, MKK4, and MKK7. Docking of these compounds into the active sites of ASK1, MKK4, and MKK7 revealed coherent binding patterns across the MAPK cascade, consistent with recognition of conserved kinase catalytic features (Figure 7). The predicted binding modes were energetically favorable (Vina scores −7.30 to −8.94 kcal/mol), with the highest affinities observed for ASK1, and suggested plausible engagement of hinge motifs, β_3_-lysine catalytic regions, hydrophobic shells, and the Asp–Phe–Gly (DFG) motif.

In the case of ASK1, apigenin showed the lowest predicted docking energy (−8.942 kcal/mol), followed closely by luteolin (−8.890 kcal/mol), while rosmarinic acid displayed a slightly weaker, though still favorable, score (−8.205 kcal/mol). All three ligands made contact with Val757, an important hinge residue that anchors ATP-competitive inhibitors within the co-crystal structure [28], with apigenin and rosmarinic acid also interacting with Gly759. Additional contacts of luteolin and apigenin with Lys688 (near the conserved β3 catalytic lysine, Lys709) were consistent with positioning within the adenine pocket, whereas rosmarinic acid extended its contact network into the rear polar pocket via Ser761, Ala764, and Asp822, suggestive of engagement with the back-pocket region described for ASK1 [28].

In MKK4, the three compounds exhibited similar predicted binding energies (−8.011 to −8.334 kcal/mol). Luteolin interacted with Ile108, Ala112, and Ala129, indicating occupation of the hydrophobic shell and hinge region (Ala112) within the active site [29]. Apigenin and rosmarinic acid formed contacts with Met178, Glu179, Met181, and Ser184, residues adjacent to the activation segment and acidic activation loop cluster (Asp186, Asp229, Asp247), suggesting that these ligands may occupy both the hinge-proximal pocket and regions near the catalytic/activation segment [29].

Binding energies were moderately weaker for MKK7 (−7.308 to −7.610 kcal/mol) (Table 4). Luteolin and apigenin mainly interacted with Ile210, Met212, and Met215, which lie within the hinge–gatekeeper environment shaping the ATP-binding cleft [30]. Moreover, rosmarinic acid made contact with Glu213 and Met215, residues of the DFG motif (Asp277, Gly279), and Ser144.

Overall, these molecular docking results indicate that luteolin, apigenin, and rosmarinic acid can potentially occupy ATP-competitive pockets of ASK1, MKK4, and MKK7 and form interactions consistent with known kinase active-site architectures. In this model, flavones (luteolin and apigenin) tend to engage conserved hinge and β-sheet elements, whereas rosmarinic acid may preferentially extend toward back-pocket and activation-segment residues, indicating potential differences in binding modes along the ASK1–MKK4–MKK7 axis. These in silico findings are concordant with the observed attenuation of JNK activation and partial preservation of β-catenin-linked osteogenic signaling in WGE-treated cells, but they should be regarded as predictive and hypothesis-generating, as direct kinase activity assays and pathway-specific inhibitor studies were not performed in this work.

## 3. Discussion

Bisphenol A (BPA), one of the most pervasive endocrine-disrupting chemicals, has been consistently associated with skeletal fragility and osteogenic impairment through multifactorial mechanisms involving oxidative stress, mitochondrial injury, and hormonal interference [6,31]. This study demonstrates, for the first time, that ethanolic extract of *Wolffia globosa* (WGE) confers significant protection against BPA-induced osteoblast dysfunction by coordinating a multi-level cytoprotective response. Treatment with WGE reduced oxidative stress, attenuated apoptosis and necrosis, modulated autophagy-related markers and stress signaling, and partially preserved osteogenic differentiation in MC3T3-E1 cells exposed to BPA. These findings support the idea that the natural agent WGE is potentially capable of alleviating the adverse effects of environmental endocrine disruptors on osteogenic cells.

The protective effects of WGE appear to result from its capacity to modulate several interconnected pathways critical for bone formation. In the acute model, WGE decreased BPA-induced ROS accumulation while also inducing expression of the antioxidant enzymes HO-1 and SOD-1 at non-cytotoxic concentrations, consistent with both direct radical scavenging and enhancement of endogenous redox defenses. In parallel, WGE significantly reduced the levels of BPA-induced apoptosis and PI-positive cell death and reversed the BPA-driven increase in cleaved caspase-3 and PARP with restoration of procaspase-3 levels [24,32,33]. Together, these data indicate that WGE limits oxidative damage and activation of caspase-dependent cell death, thereby preserving osteoblast viability under acute BPA stress. These observations are in line with, and extend, previous reports describing antioxidant and cytoprotective properties of *Wolffia*-derived or related plant extracts [12,19,25,27].

WGE also influenced the activity of autophagy-related markers and stress-responsive signaling pathways caused by BPA. Acute BPA exposure increased p62, reduced Beclin-1, and lowered the LC3-II/LC3-I ratio, while decreasing pAkt/Akt and increasing pJNK/JNK, findings consistent with a disrupted autophagy-related response and the activation of stress kinases [34,35]. WGE pretreatment reduced p62, increased Beclin-1 and LC3-II/LC3-I at selected doses, partially restored pAkt/Akt, and attenuated pJNK/JNK phosphorylation without inducing additional cytotoxicity [36,37,38]. Although autophagic flux was not directly assessed with lysosomal inhibitors, these coordinated changes suggest that WGE may help rebalance stress-responsive pathways that interface with autophagy under BPA challenge [38], rather than definitively restoring autophagic flux. This interpretation is consistent with the concept that the fine-tuning of PI3K/Akt and JNK signaling can support adaptive autophagy and survival in osteoblasts exposed to oxidative insults [6,14,38,39].

In the chronic low-dose model, WGE partially counteracted the inhibitory effects of BPA on osteogenic differentiation. BPA reduced ALP staining, mineralized nodule formation, and the expression of early osteogenic genes (*Runx2*, *Col1a1*, *ALP*), while co-treatment with WGE restored these readings toward control levels and enhanced OCN expression at 25 µg/mL, consistent with improved progression to later differentiation stages. At the signaling level, BPA caused a modest decrease in the protein β-catenin and the p-GSK3β/GSK3β ratio, whereas WGE induced a partial increase in both parameters at specific concentrations. These effects suggest that WGE can mitigate the BPA-mediated suppression of Wnt/β-catenin-linked osteogenic programs, although the magnitude of β-catenin and p-GSK3β changes is moderate and should be interpreted as supportive rather than definitive evidence of pathway reactivation. Assessment of osteogenic markers and mineralization at additional later time points would further clarify the durability of the impact of WGE on late-stage osteoblast maturation. These findings complement previous research indicating that environmental contaminants such as BPA impair bone integrity by affecting crucial molecular regulators [39,40]. The medicinal potential of bioactive substances obtained from plants in minimizing these negative impacts was also emphasized in [41,42].

Beyond the in vitro protective effects, the results of this study could emphasize the promising potential of WGE as a natural agent for combating bone fragility induced by environmental pollutants such as BPA. Given the widespread human exposure to BPA and its recognized deleterious effects on bone metabolism, the ability of WGE to modulate critical signaling pathways, reduce oxidative damage, and restore osteoblast differentiation raises the possibility that WGE might have translational relevance that warrants evaluation in in vivo models. The antioxidant-rich profile of WGE, encompassing flavonoids, phenolics, and other bioactive compounds, likely underpins these multifactorial benefits. The phytochemical profiling of WGE measured by LC-MS/MS revealed luteolin as the predominant bioactive compound, accompanied by significant amounts of rosmarinic acid and apigenin. The presence of these flavonoids and phenolic acids is particularly interesting due to their extensively documented biological activities involving oxidative stress, inflammation, and bone metabolism [43,44,45,46].

Luteolin, found in WGE at 116.17 ± 0.69 µg/g extract, has been extensively studied for its potent antioxidant, anti-inflammatory, and anti-apoptotic properties [47,48,49]. Previous studies have demonstrated its ability to scavenge reactive oxygen species, inhibit pro-inflammatory signaling pathways such as NF-κB and MAPKs, and promote osteogenic differentiation via modulation of Wnt/β-catenin signaling [50,51]. Luteolin has also been shown to promote osteoblast proliferation and mineralization while inhibiting osteoclastogenesis, therefore contributing to bone homeostasis and protecting against bone-resorptive diseases. Rosmarinic acid, a phenolic acid also detected in considerable amounts in WGE (54.80 ± 2.12 µg/g extract), is recognized as having strong radical scavenging activity and the capacity to modulate inflammation and apoptosis [52,53]. Its role in the promotion of osteoblast viability and differentiation, primarily through antioxidant mechanisms and the attenuation of oxidative stress-mediated damage, has been reported [54]. Rosmarinic acid has been shown to enhance bone matrix synthesis and mineral deposition in in vitro and in vivo models [55,56]. Apigenin, present at a similar level (48.77 ± 0.61 µg/g) to rosmarinic acid in WGE, is a bioactive flavonoid recognized for its beneficial properties in bone health [57,58]. Its antioxidant and anti-inflammatory effects complement its ability to stimulate the expression of osteogenic genes and suppress the differentiation of osteoclasts via regulatory pathways including NF-κB and MAPK signaling [59,60,61]. Several studies have indicated its efficacy in enhancing bone density and reducing bone loss in osteoporosis models.

The levels of luteolin, rosmarinic acid, and apigenin detected in WGE are within or close to ranges that have been reported as exerting biological activities relevant to osteoblast protection and bone health [62,63]. Concentrations of luteolin, rosmarinic acid, and apigenin in the range of ~30–100 µM have been shown to exhibit potent antioxidant, anti-inflammatory, and osteogenic effects, including stimulation of osteoblast proliferation and differentiation via modulation of Wnt/β-catenin signaling and anti-osteoclastogenic activity, contributing to bone Homeostasis [64,65]. Considering the measured content of luteolin (~116 µg/g), rosmarinic acid (~55 µg/g), and apigenin (~49 µg/g) in WGE and the proportion of the extract dosing in cell culture assays, it is probable that the concentrations of these compounds in the experimental setup are sufficient to elicit the observed protective effects. Additionally, synergistic interactions between these phytochemicals may enhance the overall level of efficacy beyond that of individual compounds alone. The phytochemical composition of WGE measured in this study provides a strong mechanistic justification for the observed cytoprotective effects against BPA-induced osteoblast dysfunction of the extract. The synergy between luteolin, rosmarinic acid, and apigenin may enhance the antioxidant capacity, anti-apoptotic action, and the level of promotion of osteogenesis observed in this study, confirming and extending findings from related research in the field. This phytochemical profile underlines the therapeutic potential of WGE as a multifaceted nutraceutical targeting bone health under toxic environmental exposures. Nevertheless, although the antioxidant potential of these individual constituents has been well established in previous studies, future work will be needed to dissect their direct quantitative contributions to the overall antioxidant effect of WGE in a BPA-exposed osteoblast model.

The broad protective actions of WGE observed in this study may also be relevant to skeletal changes associated with pollutant-accelerated aging. Osteoblast dysfunction driven by chronic environmental exposures contributes to age-related bone fragility and disorders such as osteoporosis [66], in part through redox imbalance and impaired stress-response pathways [67,68]. By the attenuation of intracellular ROS, the enhancing of endogenous antioxidant defenses, the limiting of apoptosis and necrosis, and the modulation of autophagy-related markers and associated signaling, WGE appears to help in the preservation of osteoblast integrity under BPA challenge. Partial support of Wnt/β-catenin-linked osteogenesis and osteogenic differentiation may further contribute to the maintenance of bone remodeling capacity, which typically declines with age [69,70]. Considering the worldwide increase in pollutant exposure, especially in occupational groups (e.g., plastic workers and cashiers who have been shown to have serum BPA levels several-fold higher than the general population) [71,72], postmenopausal women, and an aging population susceptible to diminished bone health, WGE constitutes a promising natural intervention candidate to alleviate pollution-associated osteosenescence. Our use of 50 µM for acute mechanistic studies and 10 µM for chronic differentiation assays falls within the range commonly employed in osteogenic and systemic BPA models (Table S1), which capture tissue-level and experimental exposures higher than typical serum levels but are widely used for mechanistic interrogation. Nonetheless, these conclusions are based on in vitro MC3T3-E1 models, and any potential role of WGE in mitigating pollution-associated osteosenescence remains speculative; rigorous in vivo studies in appropriate aging and exposure models, together with pharmacokinetic, bioavailability, and safety evaluations, will be essential before translational or anti-aging applications can be considered.

## 4. Materials and Methods

### 4.1. Chemicals and Reagents

Bisphenol A (BPA; ≥99% purity), dimethyl sulfoxide (DMSO), and 3-(4,5-dimethylthiazol-2-yl)-2,5-diphenyltetrazolium bromide (MTT) were purchased from Sigma-Aldrich (St. Louis, MO, USA). Antibodies against LC3B, Beclin-1, caspase-3, β-catenin, p-GSK3β, and β-actin were obtained from Cell Signaling Technology (Danvers, MA, USA). α-Minimum essential medium (α-MEM), penicillin-streptomycin, trypsin-EDTA, and phosphate-buffered saline (PBS) were purchased from Gibco (Thermo Fisher Scientific, Waltham, MA, USA). Fetal bovine serum (FBS) was obtained from HyClone Laboratories (Logan, UT, USA). All other reagents were of analytical grade unless otherwise specified.

### 4.2. Preparation of Wolffia globosa Ethanolic Extract

Freeze-dried organic *Wolffia globosa* (WG) was purchased from an organic farm located in Bang Pa-in sub-district, Phra Nakhon Si Ayutthaya, Thailand. The dried material was ground into powder and subjected to extraction with 80% ethanol at a solid-to-solvent ratio of 1:30 (*w*/*v*) overnight with intermittent stirring. This extraction procedure was repeated five times to maximize yield. The combined ethanol extracts were filtered and concentrated using a rotary evaporator, followed by freeze-drying using a lyophilizer (Beta 2–8 Cbasic, CHRIST, Osterode am Harz, Germany). The yield of the ethanol extract (WGE) was 18.5%. All dried extracts were stored at −20 °C until use. Dried WGE was redissolved in DMSO to prepare stock solutions. The final DMSO concentration in all treatments did not exceed 0.1% (*v*/*v*).

### 4.3. Measurement of Total Phenolic and Flavonoid Contents

Total phenolic content was analyzed via the Folin–Ciocalteu colorimetric assay in accordance with the method described by Singleton and Rossi [73], with minor modifications. The phenolic compounds in the extract reduce the Folin–Ciocalteu reagent (Merck KGaA, Darmstadt, Germany), resulting in a color change proportional to the phenolic concentration. The reaction mixture was incubated in the dark for 30 min before absorbance measurement at 765 nm. Gallic acid was used to generate a standard curve, allowing phenolic content to be expressed as milligrams of gallic acid equivalence (GAE) per gram of extract.

Total flavonoid content was determined using an aluminum chloride colorimetric assay, adapted with slight modifications from previously established protocols [74]. In this method, flavonoids present in the extract form stable complexes with aluminum chloride (AlCl_3_) Sigma-Aldrich (St. Louis, MO, USA), producing a measurable color change. Briefly, aliquots of the extract were mixed with 2% AlCl_3_ solution and incubated for 60 min at room temperature. The absorbance of the resulting complex was measured at 420 nm using a UV-Vis spectrophotometer (BioTek Synergy/H1 microplate reader (Agilent, Santa Clara, CA, USA). Quantification was achieved by comparison to a standard calibration curve generated with catechin, and results were expressed as milligrams of catechin equivalents (CE) per gram of extract. All measurements were performed in triplicate to ensure accuracy and reliability of the data.

### 4.4. Targeted Phytochemical Analysis by LC-MS/MS

Liquid chromatography–tandem mass spectrometry (LC-MS/MS) was used to identify bioactive metabolites present in WGE. A high-resolution LC-MS/MS system with an electrospray ionization (ESI) source was operated in both positive and negative ion modes. Chromatographic separation was performed using a 2.1 mm × 100 mm, 2.6 μm Accucore RP-MS (Thermo Fisher Scientific, Bremen, Germany) reversed-phase column under optimized gradient conditions with a binary solvent system: Acetonitrile (Solvent A) and Milli-Q water (18.2 MΩ cm resistivity at 25 °C) containing 0.1% formic acid (Solvent B). Flow rate (0.5 mL/min), injection volume (10 µL), running time (10 min), and gradient program were optimized to maximize peak resolution and metabolite detection. Mass spectra were acquired in multiple reaction monitoring (MRM) and full-scan modes. Data analysis was performed using instrument-specific software. Metabolite identification was based on comparison of retention times and mass fragmentation patterns with twenty-four authenticated reference standards obtained from Sigma-Aldrich (St. Louis, MO, USA), including 3,4-dihydroxybenzoic acid, 4-hydroxybenzoic acid, apigenin, caffeic acid, chlorogenic acid, cinnamic acid, epicatechin gallate, ferulic acid, galangin, gallic acid, genistein, hesperidin, isorhamnetin, kaempferol, luteolin, myricetin, naringenin, p-coumaric acid, quercetin, rosmarinic acid, rutin, sinapic acid, syringic acid, and vanillic acid, as well as spectral databases.

### 4.5. In Vitro Antioxidant Activity Measurement

The radical scavenging activity of 2,2′-azinobis-(3-ethylbenzothiazoline-6-sulfonic acid) (ABTS) (Sigma-Aldrich, St. Louis, MO, USA) was determined following the method described in a previous study [75]. The 2,2-diphenyl-1-picrylhydrazyl (DPPH) assay (Sigma-Aldrich, St. Lou-is, MO, USA) was performed in accordance with the procedure reported in an earlier study [76], with slight modifications. Antioxidant activities were expressed as % inhibition using the equation below.

(1) $$ABTS\cdot +/DPPH\;scavenging\;effect\;(\%)=\left[\frac{\mathrm{Abs}\;\mathrm{control}-\mathrm{Abs}\;\mathrm{extract}}{\mathrm{Abs}\;\mathrm{control}}\right]\times 100$$

The antioxidant capacities of ABTS·+ and DPPH radicals were reported as SC_50_ values, corresponding to the extract concentration (µg/mL) that produces 50% scavenging of the radicals.

The ferric reducing antioxidant power (FRAP) (Sigma-Aldrich, St. Louis, MO, USA) of the samples was determined as previously described with slight modification [77,78]. The FRAP reagent was freshly prepared by combining 2.5 mL of 10 mM TPTZ solution in 40 mM HCl, 2.5 mL of a 20 mM FeCl_3_·6H_2_O solution, and 20 mL of a 300 mM acetate buffer (pH 3.6). This solution was incubated at 37 °C for 30 min before use. Subsequently, 50 μL of the WGE sample was added to 750 μL of the FRAP reagent and left in the dark for 30 min. The increase in color was measured at a wavelength of 595 nm using a spectrophotometer. The results were reported as mmol of FeSO_4_ per g DW of sample.

The oxygen radical absorbance capacity (ORAC) assay was performed by mixing 200 μL of WGE with 1.6 mL of 0.04 μM β-phycoerythrin (prepared in 0.075 M sodium phosphate buffer (pH 7.0)). The mixture was incubated in the dark at 37 °C for 10 min, followed by the addition of 200 μL of 4 mM AAPH (Sigma-Aldrich, St. Louis, MO, USA) to initiate peroxyl radical generation. The fluorescence decay was then observed at an emission wavelength of 528 nm and an excitation wavelength of 485 nm at 37 °C every 5 min using a spectrophotometer (BioTek Synergy/H1 microplate reader (Agilent, Santa Clara, CA, USA). Trolox was employed as the standard, and results were reported as μM Trolox equivalence (TE) per g dry weight of sample [78].

### 4.6. Cell Culture and Treatment

The MC3T3-E1 murine pre-osteoblast cell line (subclone 4) was obtained from the American Type Culture Collection (ATCC, Manassas, VA, USA). The cells were cultured in α-MEM supplemented with 10% FBS and 1% penicillin-streptomycin at 37 °C in a humidified 5% CO_2_ incubator and were passaged when they reached 70–80% confluence. The cells at passages 3–10 were used for all experiments.

In the present study, BPA concentrations were selected based on established in vitro and in vivo toxicological precedents. Table S1 compiles 20 peer-reviewed osteogenic and non-osteogenic studies employing 1–50 μM BPA for investigating oxidative stress, apoptosis, cellular dysfunction, and osteogenic impairment, confirming the methodological validity of our experimental design.

For the treatments, two exposure models were established, including acute or short-term exposure and chronic or long-term exposure. An acute high-dose BPA or short-term exposure model (50 µM, 12–24 h) was specifically designed to induce reproducible oxidative stress, apoptosis, and signaling perturbations in MC3T3-E1 osteoblasts, facilitating a thorough mechanistic analysis of WGE’s cytoprotective pathways. MC3T3-E1 cells were seeded in 6-well plates at a density of 4 × 10^4^ cells/cm^2^ and allowed to adhere overnight. The cells were either treated with BPA or WGE alone or pretreated with WGE (0–50 μg/mL) for 2 h, followed by co-treatment with BPA (50 μM) for 12 or 24 h. After the short-term treatment, the cells were collected and subjected to the measurement of cell viability, intracellular ROS production, cell death and apoptosis, and autophagy marker levels. Chronic exposure applied during osteogenic differentiation was used to determine the reverse effect of WGE on osteogenic differentiation suppressed by BPA. The cells were cultured in an osteogenic medium containing α-MEM supplemented with 5 mM β-glycerophosphate, 100 μg/mL ascorbic acid (Sigma-Aldrich, St. Louis, MO, USA), and 10 nM dexamethasone (Sigma-Aldrich, St. Louis, MO, USA). BPA (10 μM) in the presence or absence of WGE (12.5, 25 μg/mL) was added throughout the differentiation period. The medium was replaced every 2–3 days. At designated endpoints (days 7, 14 and 21), osteogenic differentiation was evaluated. A 0.1% DMSO vehicle control (without WGE or BPA) was included in all experiments and is the “control” group referenced throughout.

### 4.7. Cell Viability Assay

The 3-(4,5-dimethylthiazol-2-yl)-2,5-diphenyltetrazolium bromide (MTT) assay was used to determine cell viability. MC3T3-E1 cells were seeded in 96-well plates and treated with various concentrations of WGE (6.25–200 μg/mL) for 24 h. MTT (0.5 mg/mL) was added, and then the cells were incubated for 4 h, followed by solubilization with DMSO. Absorbance of the formazan was measured at 570 nm. Using a microplate reader.

(2) $$Viability\;(\%)=\left[100\times \frac{{\mathrm{Abs}}_{570}\;\mathrm{Sample}}{{\mathrm{Abs}}_{570}\;\mathrm{Blank}\;(\mathrm{Control})}\right]$$

### 4.8. Flow Cytometry for Apoptosis

Apoptosis was measured using an Annexin V/PI apoptosis detection kit (Sigma-Aldrich). After treatment, cells were harvested, washed, and resuspended in binding buffers. Then 5 µL of Annexin V-FITC was added and incubated for 5 min at 2–8 °C in the dark. After that, 10 µL of propidium iodide was added, and the cell suspension was incubated for 5 min at 2–8 °C in the dark. Finally, cells were analyzed by flow cytometry (BD FACS Vantage SE); Annexin V-positive/PI-positive cells were considered late apoptotic or necrotic cells.

### 4.9. H_2_DCFDA Assay

Intracellular ROS level was detected using 2′,7′-dichlorodihydrofluorescein diacetate (H_2_DCFDA; Thermo Fisher). After treatment, the cells were incubated with 10 μM H_2_DCFDA at 37 °C for 30 min. Fluorescence intensity was imaged via fluorescence microscopy and quantified using a microplate reader (excitation: 485 nm; emission: 530 nm).

### 4.10. Western Blotting

The proteins were extracted using RIPA buffer with protease and phosphatase inhibitors, separated by SDS-PAGE, and subsequently transferred to nitrocellulose membranes. The membrane underwent incubation with 5% BSA in Tris-buffered saline (TBS) for 1 h at room temperature to inhibit non-specific binding. The samples were subsequently incubated with a primary antibody diluted in 5% BSA in TBS overnight at 4 °C. Following washing, the membranes underwent incubation with a secondary antibody conjugated to HRP. The membranes were analyzed using enhanced chemiluminescence substrate, and detection was carried out using a gel documentation imaging system. Protein band intensity was quantified using ImageJ software (version 1.54g). Proteins were detected using antibodies recognizing LC-3I/LC-3II (18/14 kDa), Beclin-1 (60 kDa), p62 (62 kDa), β-catenin (92 kDa), p-Akt/Akt (62 kDa), p-JNK/JNK (46/54 kDa), SOD-1 (16 kDa), HO-1 (32 kDa), PARP (116 kDa, cleaved 89 kDa), and caspase-3 (32 kDa, cleaved 17/19 kDa), with β-actin (42 kDa) as the loading control. All immunoblot experiments were performed using at least three independent biological replicates (independent cell culture and treatment experiments), with representative blots shown in the main figures.

### 4.11. Osteogenic Differentiation Assays

MC3T3-E1 cells were cultured in osteogenic medium (α-MEM + 100 µg/mL ascorbic acid + 5 mM β-glycerophosphate and 10 nM dexamethasone) for 7, 14, and 21 days in the presence or absence of BPA (10 μM) with or without WGE (12.5 and 25 μg/mL).

Alkaline Phosphatase (ALP) Activity: ALP activity was quantified on day 7 using a p-nitrophenyl phosphate (pNPP)-based colorimetric assay (Sigma-Aldrich, St. Louis, MO, USA) as previously described [79]. The reaction product (p-nitrophenol) was measured at 405 nm. Activity was normalized to total protein content and expressed as U/g protein.

ALP Staining: The cells were fixed and stained for the identification of alkaline phosphatase (ALP) on day 14 using a commercial ALP staining kit (Merck KGaA, Darmstadt, Germany), in accordance with the manufacturer’s instructions [80]. Stained cultures were observed and imaged under a light microscope.

Alizarin Red S Staining: After 21 days of osteogenic induction, calcium deposition was evaluated using Alizarin Red S staining (Sigma-Aldrich, St. Louis, MO, USA). Cells were washed twice with PBS and fixed with 4% paraformaldehyde for 15 min at room temperature. Fixed cells were then stained with 2% Alizarin Red S solution (pH 4.2) for 30 min with gentle agitation. Excess dye was removed by rinsing with deionized water. For quantitative analysis, the bound dye was extracted using 10% cetylpyridinium chloride (CPC) in distilled water for 30 min at room temperature. The solubilized dye was collected, and absorbance was measured at 562 nm using a microplate reader (BioTek Synergy H1). The amount of extracted Alizarin Red S correlated with the degree of mineralization is expressed as relative absorbance normalized to the control group [81].

mRNA Expression by RT-qPCR: After treatment for 14 days, total RNA was isolated from the treated cells using an Omega RNA Purification Kit (Omega Bio-tek, Norcross, GA, USA). Subsequently, 1 μg of RNA was reverse transcribed into cDNA using a Bioline cDNA Synthesis Kit (Bioline, London, UK). Quantitative PCR was performed using an Applied Biosystems 7500/7500 Fast Real-Time PCR System using SYBR^®^ Green qPCR Master Mix with ROX (Solis BioDyne, Tartu, Estonia) for 45 amplification cycles. Each cycle consisted of 5 s at 95 °C, 10 s at 60 °C, and 30 s at 72 °C. The gene expression level of *Runx2*, *Col1a*1, *OCN*, and *ALP* was normalized to the *GAPDH* reference gene and quantified using the 2^−ΔΔCt^ method. Primer sequences are shown in Table 5.

### 4.12. Molecular Docking Analysis

Since insufficient mouse protein structures were available, the crystallographic structures of human ASK1, MKK4, and MKK7 retrieved from the NCBI Protein Data Bank (PDB IDs: 3VW6, 3ALN, and 6YFZ, respectively) were used as docking templates. The kinase catalytic domains and ATP-binding/active-site residues of these MAP3K/MAP2K family members are highly conserved in similarity between human and mouse orthologs [82]. The structures of the potential active compounds (luteolin, apigenin, and rosmarinic acid) were obtained from PubChem. Before performing molecular docking in Chimera 1.17.3, AutoDock Tools 1.5.6 and MGLTools 1.5.6 were employed to remove water molecules and add the necessary hydrogen atoms. The grid box (Å) and size box were set at 6.5, 5.2, 25.5 and 57, 30, 25 for ASK1, 16.0, 2.0, −23.0, and 54, 23, 26 for MKK4 and 1.5, 31.0, 10.0 and 25, 29, 25 for MKK7. Molecular visualization and interaction analysis were conducted using Discovery Studio 3.0.

### 4.13. Statistical Analysis

All data were obtained from at least three independent experiments and expressed as mean ± SD. Statistical significance was assessed using one-way ANOVA followed by Tukey’s post hoc test (*p* < 0.05 considered significant). All analyses were performed using GraphPad Prism 10.4.2 (GraphPad Software, CA, USA).

## 5. Conclusions

In this study, we demonstrated that *Wolffia globosa* ethanolic extract (WGE) protects MC3T3-E1 osteoblasts from bisphenol A (BPA)-induced toxicity in vitro through coordinated actions in multiple cellular pathways. WGE enhanced antioxidant defenses and suppressed the accumulation of BPA-induced reactive oxygen, upregulated cytoprotective enzymes such as HO-1 and SOD-1, and reduced apoptosis and necrosis by attenuating the activation of caspase-3 and PARP. WGE also modulated autophagy-related markers, including LC3, Beclin-1, and p62, and partially restored the balance of Akt/JNK signaling, suggesting a broader role in supporting cellular stress-response homeostasis under BPA challenges. Under chronic low-dose exposure, WGE improved alkaline phosphatase activity, mineralized nodule formation, and the expression of the osteogenic markers Runx2, Col1a1, ALP, and OCN, accompanied by a partial recovery of β-catenin signaling, thereby supporting osteoblast differentiation and matrix mineralization.

Phytochemical analysis indicated that WGE is notably enriched in luteolin, rosmarinic acid, and apigenin, which likely contribute to these antioxidant, anti-apoptotic, and pro-osteogenic effects through complementary and potentially synergistic mechanisms. Together, these in vitro findings suggest that WGE could represent a sustainable nutraceutical candidate for the mitigation of pollutant-related bone deterioration and that its multitargeted actions may have relevance for broader anti-aging strategies in environments with increasing chemical stress. However, future in vivo, translational, and clinical studies are warranted to establish the efficacy, safety, and optimal application of WGE in the prevention of bone fragility and age-related skeletal decline associated with chronic exposure to environmental toxins.

### Limitation

This study has several limitations which need to be acknowledged. First, although BPA and WGE induced consistent changes in LC3-II, Beclin-1, and p62, autophagic flux was not directly measured using lysosomal inhibitors; therefore, conclusions regarding autophagy remain inferential, and the observed effects should be regarded as modulation of autophagy-related markers rather than proven restoration of flux. Second, the docking analysis of luteolin, apigenin, and rosmarinic acid with ASK1, MKK4, and MKK7 was performed solely in silico and was not followed by biochemical validation of kinase activity or downstream phosphorylation and, therefore, serves as hypothesis-generating rather than definitive evidence. Third, acute high-dose (50 µM, 12–24 h) and chronic low-dose (10 µM, 7–21 days) BPA models were used to dissect short-term stress responses and long-term osteogenic impairment, but ROS, apoptosis, and autophagy-related markers were not assessed during the chronic phase. Finally, although all Western blot data are based on at least three independent biological experiments with densitometric quantification, some variability was observed between replicates, particularly in the case of autophagy-related proteins, underscoring the need for additional confirmatory studies in complementary systems.

## Figures and Tables

**Table 1:** Extraction Yield, Total Phenolic and Flavonoid Content, and Antioxidant Activity of WGE.

Parameter	WGE
Extraction yield (%)	18.75
Total phenolic content (mg GAE/g extract)	42.82 ± 3.56
Total flavonoid content (mg CE/g extract)	17.79 ± 1.96

*Data represented as mean ± SD (n = 3).*

**Table 2:** Rosmarinic Acid, Luteolin, and Apigenin Contents in WGE Determined by LC–MS.

Sample	Rosmarinic Acid (µg/g Extract)	Luteolin (µg/g Extract)	Apigenin (µg/g Extract)
WGE	54.80 ± 2.12	116.17 ± 0.69	48.77 ± 0.61

*Data represented as mean ± SD (n = 3).*

**Table 3:** Antioxidant Activity of WGE.

Parameter	WGE
ABTS scavenging activity (SC_50_; µg/mL)	40.47 ± 7.50
DPPH scavenging activity (SC_50_; µg/mL)	247.27 ± 33.81
ORAC (µmol Trolox/g extract)	1725.85 ± 99.76
FRAP (µmol Trolox/g extract)	210.07 ± 2.62

*Data represented as mean ± SD (n = 3).*

**Table 4:** Vina Scores (Predicted Binding Affinities), The Number of Hydrogen Bonds, and the Interacting Amino Acid Residues of ASK1, MKK4, and MKK7 for Luteolin, Apigenin, and Rosmarinic Acid.

Chemical Compounds	Vina Score (kcal/mol)	H-Bond	Interacting Amino Acid Residues
**ASK1 (PDB: 3VW6)**			
Luteolin	−8.890	2	LYS688, LEU686, VAL757
Apigenin	−8.942	2	LYS688, GLY759, VAL757
Rosmarinic acid	−8.205	1	VAL757, GLY759, ASP822, SER761, ALA764
**MKK4 (PDB: 3ALN)**			
Luteolin	−8.011	2	ILE108, ALA112, ALA129, MET181
Apigenin	−8.247	3	MET178, GLU179, ALA129, MET181, SER184, ARG110, TYR119
Rosmarinic acid	−8.334	2	SER184, MET181, GLU179, MET178, TYR113
**MKK7 (PDB: 6YFZ)**			
Luteolin	−7.610	1	SER263, ILE210, MET215, MET212
Apigenin	−7.355	1	MET215, ILE210
Rosmarinic acid	−7.308	2	GLU213, MET215, ASP277, GLY279, SER144

**Table 5:** Primer Sequences for Quantitative RT-qPCR.

Gene	Forward Primer	Reverse Primer
*Runx2*	TGGCCGGGAATGATGAGAAC	TGAAACTCTTGCCTCGTCCG
*ALP*	CACTCTGTCCCGTTGGTGTC	TTGACGTTCCGATCCTGCAC
*OCN*	CAGACCTAGCAGACACCATGAGG	AGGTCAGAGAGACAGAGCGCA
*Col1a1*	TTCTCCTGGCAAAGACGGAC	CTCAAGGTCACGGTCACGAA
*GAPDH*	TGGCAAAGTGGAGATTGTTGCC	AAGATGGTGATGGGCTTCCCG

## Data Availability

The original contributions presented in this study are included in the article/Supplementary Materials. Further inquiries can be directed to the corresponding author(s).

## Supplementary Materials

The following supporting information can be downloaded at: https://www.mdpi.com/article/10.3390/ijms27125352/s1. References [5,39,40,83,84,85,86,87,88,89,90,91,92,93,94,95,96,97,98,99] are cited in the supplementary materials.

## Abbreviations

The following abbreviations are used in this manuscript:

ABTS	2,2′-Azino-bis(3-ethylbenzothiazoline-6-sulfonic acid)
ALP	Alkaline phosphatase
ASK1	Apoptosis signal-regulating kinase 1
BPA	Bisphenol A
CE	Catechin equivalents
Col1a1	Collagen type I alpha 1
DCF-DA	2′,7′-Dichlorodihydrofluorescein diacetate
DMSO	Dimethyl sulfoxide
DPPH	2,2-Diphenyl-1-picrylhydrazyl
EDCs	Endocrine-disrupting chemicals
FRAP	Ferric reducing antioxidant power
GAE	Gallic acid equivalents
GSK3β	Glycogen synthase kinase 3 beta
HO-1	Heme oxygenase-1
IC_50_	Half-maximal inhibitory concentration
JNK	c-Jun N-terminal kinase
LC3	Microtubule-associated protein 1A/1B-light chain 3
LC-MS/MS	Liquid chromatography–tandem mass spectrometry
MFI	Mean fluorescence intensity
MKK4	Mitogen-activated protein kinase kinase 4
MKK7	Mitogen-activated protein kinase kinase 7
MTT	3-(4,5-Dimethylthiazol-2-yl)-2,5-diphenyltetrazolium bromide
OCN	Osteocalcin
ORAC	Oxygen radical absorbance capacity
P62	Nuclear pore glycoprotein p62
PARP	Poly (ADP-ribose) polymerase
PI	Propidium iodide
PI3K	Phosphoinositide 3-kinase
ROS	Reactive oxygen species
Runx2	Runt-related transcription factor 2
SC_50_	Half-maximal scavenging concentration
SOD-1	Superoxide dismutase 1
WG	*Wolffia globosa*
WGE	*Wolffia globosa* ethanolic extract

## References

[1] Compston J. (2020). Practical guidance for the use of bisphosphonates in osteoporosis. Bone.

[2] Pagnotti G.M., Thompson W.R., Guise T.A., Rubin C.T. (2021). Suppression of cancer-associated bone loss through dynamic mechanical loading. Bone.

[3] Kumar M., Xiong X., He M., Tsang D.C.W., Gupta J., Khan E., Harrad S., Hou D., Ok Y.S., Bolan N.S. (2020). Microplastics as pollutants in agricultural soils. Environ. Pollut..

[4] Jiang W., Ding K., Huang W., Xu F., Lei M., Yue R. (2023). Potential effects of bisphenol A on diabetes mellitus and its chronic complications: A narrative review. Heliyon.

[5] Shi X., Wu K., Liu C., Cao K., Zhang Q., Wu W., Luo C., Huang W. (2024). Preliminary investigation into the impact of BPA on osteoblast activity and bone development: In vitro and in vivo models. Environ. Pollut..

[6] Chin K.Y., Pang K.L., Mark-Lee W.F. (2018). A Review on the Effects of Bisphenol A and Its Derivatives on Skeletal Health. Int. J. Med. Sci..

[7] Moreno-Gomez-Toledano R., Sanchez-Esteban S., Cook A., Minguez-Moratinos M., Ramirez-Carracedo R., Reventun P., Delgado-Marin M., Bosch R.J., Saura M. (2021). Bisphenol A Induces Accelerated Cell Aging in Murine Endothelium. Biomolecules.

[8] Khan N.G., Tungekar B., Adiga D., Chakrabarty S., Rai P.S., Kabekkodu S.P. (2023). Alterations induced by Bisphenol A on cellular organelles and potential relevance on human health. Biochim. Biophys. Acta Mol. Cell Res..

[9] Mustieles V., D’Cruz S.C., Couderq S., Rodriguez-Carrillo A., Fini J.B., Hofer T., Steffensen I.L., Dirven H., Barouki R., Olea N. (2020). Bisphenol A and its analogues: A comprehensive review to identify and prioritize effect biomarkers for human biomonitoring. Environ. Int..

[10] Gassman N.R. (2017). Induction of oxidative stress by bisphenol A and its pleiotropic effects. Environ. Mol. Mutagen..

[11] Molina-Lopez A.M., Bujalance-Reyes F., Ayala-Soldado N., Mora-Medina R., Lora-Benitez A., Moyano-Salvago R. (2023). An Overview of the Health Effects of Bisphenol A from a One Health Perspective. Animals.

[12] Meli R., Monnolo A., Annunziata C., Pirozzi C., Ferrante M.C. (2020). Oxidative Stress and BPA Toxicity: An Antioxidant Approach for Male and Female Reproductive Dysfunction. Antioxidants.

[13] Nayak D., Adiga D., Khan N.G., Rai P.S., Dsouza H.S., Chakrabarty S., Gassman N.R., Kabekkodu S.P. (2022). Impact of Bisphenol A on Structure and Function of Mitochondria: A Critical Review. Rev. Environ. Contam. Toxicol..

[14] Wang W., Ma A., Zhai C., Lan W., Liu Z., Yang Z., Zhang Y., Zhu T., Yu T., Lan J. (2025). Bisphenol A and Di-n-butyl phthalate disrupt bone homeostasis bidirectionally via CD36-mediated BMSCs autophagy inhibition and exosome-promoted osteoclastogenesis. J. Hazard. Mater..

[15] Liu J., Xiao Q., Xiao J., Niu C., Li Y., Zhang X., Zhou Z., Shu G., Yin G. (2022). Wnt/β-catenin signalling: Function, biological mechanisms, and therapeutic opportunities. Signal Transduct. Target. Ther..

[16] Gao Y., Chen N., Fu Z., Zhang Q. (2023). Progress of Wnt Signaling Pathway in Osteoporosis. Biomolecules.

[17] Priddy C., Li J. (2021). The role of the Nrf2/Keap1 signaling cascade in mechanobiology and bone health. Bone Rep..

[18] Li X., Chen Y., Mao Y., Dai P., Sun X., Zhang X., Cheng H., Wang Y., Banda I., Wu G. (2020). Curcumin Protects Osteoblasts From Oxidative Stress-Induced Dysfunction via GSK3β-Nrf2 Signaling Pathway. Front. Bioeng. Biotechnol..

[19] Tipnee S., Jutiviboonsuk A., Wongtrakul P. (2017). The Bioactivity Study of Active Compounds in *Wolffia globosa* Extract for an Alternative Source of Bioactive Substances. Cosmetics.

[20] Khonkarn R., Daowtak K., Kraseasintra O., Luetragoon T., Usuwanthim K., Taynawa K., Chanphong K. (2025). Bioactive Potential of Protein Extracts Derived from Dried *Wolffia globosa* on In Vitro Antioxidant Activities and Pro-Inflammatory Cytokine Production. Molecules.

[21] Zheng Y.H., Yang J.J., Tang P.J., Zhu Y., Chen Z., She C., Chen G., Cao P., Xu X.Y. (2021). A novel Keap1 inhibitor iKeap1 activates Nrf2 signaling and ameliorates hydrogen peroxide-induced oxidative injury and apoptosis in osteoblasts. Cell Death Dis..

[22] Che J., Yang X., Jin Z., Xu C. (2023). Nrf2: A promising therapeutic target in bone-related diseases. Biomed. Pharmacother..

[23] Jomova K., Alomar S.Y., Valko R., Liska J., Nepovimova E., Kuca K., Valko M. (2025). Flavonoids and their role in oxidative stress, inflammation, and human diseases. Chem. Biol. Interact..

[24] Amjad S., Rahman M.S., Pang M.G. (2020). Role of Antioxidants in Alleviating Bisphenol A Toxicity. Biomolecules.

[25] Yadav N.K., Patel A.B., Debbarma S., Priyadarshini M.B., Priyadarshi H. (2024). Characterization of Bioactive Metabolites and Antioxidant Activities in Solid and Liquid Fractions of Fresh Duckweed (*Wolffia globosa*) Subjected to Different Cell Wall Rupture Methods. ACS Omega.

[26] Hanga-Farcas A., Miere Groza F., Filip G.A., Clichici S., Fritea L., Vicas L.G., Marian E., Pallag A., Jurca T., Filip S.M. (2023). Phytochemical Compounds Involved in the Bone Regeneration Process and Their Innovative Administration: A Systematic Review. Plants.

[27] Monthakantirat O., Chulikhit Y., Maneenet J., Khamphukdee C., Chotritthirong Y., Limsakul S., Punya T., Turapra B., Boonyarat C., Daodee S. (2022). Total Active Compounds and Mineral Contents in *Wolffia globosa*. J. Chem..

[28] Terao Y., Suzuki H., Yoshikawa M., Yashiro H., Takekawa S., Fujitani Y., Okada K., Inoue Y., Yamamoto Y., Nakagawa H. (2012). Design and biological evaluation of imidazo[1,2-a]pyridines as novel and potent ASK1 inhibitors. Bioorg. Med. Chem. Lett..

[29] Zhang W., Shahab M., Zheng G. (2025). Targeting mitogen-activated protein kinase 4 for liver regeneration through QSAR-based virtual screening and unbiased MD simulation. Int. J. Biol. Macromol..

[30] Schröder M., Tan L., Wang J., Liang Y., Gray N.S., Knapp S., Chaikuad A. (2020). Catalytic Domain Plasticity of MKK7 Reveals Structural Mechanisms of Allosteric Activation and Diverse Targeting Opportunities. Cell Chem. Biol..

[31] Prasse T., Stratos I., Niehoff A., Christ H., Heck V., Meyer C., Mittlmeier T. (2022). Bisphenol A-Related Effects on Bone Morphology and Biomechanical Properties in an Animal Model. Toxics.

[32] Park W.H. (2022). The Anti-Apoptotic Effects of Caspase Inhibitors in Propyl Gallate-Treated Lung Cancer Cells Are Related to Changes in Reactive Oxygen Species and Glutathione Levels. Molecules.

[33] Russo C., Maugeri A., Albergamo A., Dugo G., Navarra M., Cirmi S. (2023). Protective Effects of a Red Grape Juice Extract against Bisphenol A-Induced Toxicity in Human Umbilical Vein Endothelial Cells. Toxics.

[34] Anand S.K., Sharma A., Singh N., Kakkar P. (2020). Activation of autophagic flux via LKB1/AMPK/mTOR axis against xenoestrogen Bisphenol-A exposure in primary rat hepatocytes. Food Chem. Toxicol..

[35] Lin M., Hua R., Ma J., Zhou Y., Li P., Xu X., Yu Z., Quan S. (2021). Bisphenol A promotes autophagy in ovarian granulosa cells by inducing AMPK/mTOR/ULK1 signalling pathway. Environ. Int..

[36] Liu X.Q., Jiang L., Li Y.Y., Huang Y.B., Hu X.R., Zhu W., Wang X., Wu Y.G., Meng X.M., Qi X.M. (2022). Wogonin protects glomerular podocytes by targeting Bcl-2-mediated autophagy and apoptosis in diabetic kidney disease. Acta Pharmacol. Sin..

[37] Shen S., Zhou M., Huang K., Wu Y., Ma Y., Wang J., Ma J., Fan S. (2017). Blocking autophagy enhances the apoptotic effect of 18beta-glycyrrhetinic acid on human sarcoma cells via endoplasmic reticulum stress and JNK activation. Cell Death Dis..

[38] Priego A.R., Parra E.G., Mas S., Morgado-Pascual J.L., Ruiz-Ortega M., Rayego-Mateos S. (2021). Bisphenol A Modulates Autophagy and Exacerbates Chronic Kidney Damage in Mice. Int. J. Mol. Sci..

[39] Garcia-Recio E., Costela-Ruiz V.J., Melguizo-Rodriguez L., Ramos-Torrecillas J., Garcia-Martinez O., Ruiz C., de Luna-Bertos E. (2022). Repercussions of Bisphenol A on the Physiology of Human Osteoblasts. Int. J. Mol. Sci..

[40] Maduranga Karunarathne W.A.H., Choi Y.H., Park S.R., Lee C.M., Kim G.Y. (2022). Bisphenol A inhibits osteogenic activity and causes bone resorption via the activation of retinoic acid-related orphan receptor α. J. Hazard. Mater..

[41] Agas D., Lacava G., Sabbieti M.G. (2018). Bone and bone marrow disruption by endocrine-active substances. J. Cell Physiol..

[42] Sirasanagandla S.R., Al-Huseini I., Sakr H., Moqadass M., Das S., Juliana N., Abu I.F. (2022). Natural Products in Mitigation of Bisphenol A Toxicity: Future Therapeutic Use. Molecules.

[43] Yadav N., Patel A., Debbarma S., Mocherla B., Kumar G., Baidya S., Upadhyay A. (2024). Characterization of phenolic compounds in watermeal (*Wolffia globosa*) through LC-ESI-QTOF-MS/MS: Assessment of bioactive compounds, in vitro antioxidant and anti-diabetic activities following different drying methods. J. Food Meas. Charact..

[44] Lin S., Wang Y., Du J., Xu X., Zhang X. (2024). Pharmacology and mechanisms of apigenin in preventing osteoporosis. Front. Pharmacol..

[45] Mroczek J., Pikula S., Suski S., Weremiejczyk L., Biesaga M., Strzelecka-Kiliszek A. (2022). Apigenin Modulates AnxA6- and TNAP-Mediated Osteoblast Mineralization. Int. J. Mol. Sci..

[46] Jeong M.J., Lim D.S., Kim S.O., Park C., Choi Y.H., Jeong S.J. (2021). Effect of rosmarinic acid on differentiation and mineralization of MC3T3-E1 osteoblastic cells on titanium surface. Anim. Cells Syst..

[47] Ren F., Li Y., Luo H., Gao S., Jiang S., Yang J., Rao C., Chen Y., Peng C. (2024). Extraction, detection, bioactivity, and product development of luteolin: A review. Heliyon.

[48] Punia Bangar S., Kajla P., Chaudhary V., Sharma N., Ozogul F. (2023). Luteolin: A flavone with myriads of bioactivities and food applications. Food Biosci..

[49] Peng Z., Zhang W., Hong H., Liu L. (2024). Effect of luteolin on oxidative stress and inflammation in the human osteoblast cell line hFOB1.19 in an inflammatory microenvironment. BMC Pharmacol. Toxicol..

[50] Liang G., Zhao J., Pan J., Yang Y., Dou Y., Yang W., Zeng L., Liu J. (2023). Network pharmacology identifies fisetin as a treatment for osteoporosis that activates the Wnt/β-catenin signaling pathway in BMSCs. J. Orthop. Surg. Res..

[51] Zheng H., Liu J., Sun L., Meng Z. (2024). The role of N-acetylcysteine in osteogenic microenvironment for bone tissue engineering. Front. Cell Dev. Biol..

[52] Guan H., Luo W., Bao B., Cao Y., Cheng F., Yu S., Fan Q., Zhang L., Wu Q., Shan M. (2022). A Comprehensive Review of Rosmarinic Acid: From Phytochemistry to Pharmacology and Its New Insight. Molecules.

[53] Pintha K., Chaiwangyen W., Yodkeeree S., Suttajit M., Tantipaiboonwong P. (2021). Suppressive Effects of Rosmarinic Acid Rich Fraction from Perilla on Oxidative Stress, Inflammation and Metastasis Ability in A549 Cells Exposed to PM via C-Jun, P-65-Nf-Κb and Akt Signaling Pathways. Biomolecules.

[54] Othman N.M., Elhawary Y.M., Elbeltagy M.G., Badr A.E. (2023). The Effect of Rosmarinus Officinalis as a Potential Root Canal Medication on the Viability of Dental Pulp Stem Cells. J. Contemp. Dent. Pract..

[55] Lee J.W., Asai M., Jeon S.K., Iimura T., Yonezawa T., Cha B.Y., Woo J.T., Yamaguchi A. (2015). Rosmarinic acid exerts an antiosteoporotic effect in the RANKL-induced mouse model of bone loss by promotion of osteoblastic differentiation and inhibition of osteoclastic differentiation. Mol. Nutr. Food Res..

[56] Phromnoi K., Suttajit M., Saenjum C., Limtrakul Dejkriengkraikul P. (2021). Inhibitory Effect of a Rosmarinic Acid-Enriched Fraction Prepared from Nga-Mon (*Perilla frutescens*) Seed Meal on Osteoclastogenesis through the RANK Signaling Pathway. Antioxidants.

[57] D’Amico E., Pierfelice T.V., Iezzi G., Di Pietro N., Lepore S., Lorusso F., Scarano A., Pandolfi A., Piattelli A., Petrini M. (2022). Apigenin Promotes Proliferation and Mineralization of Human Osteoblasts and Up-Regulates Osteogenic Markers. Appl. Sci..

[58] Goto T., Hagiwara K., Shirai N., Yoshida K., Hagiwara H. (2015). Apigenin inhibits osteoblastogenesis and osteoclastogenesis and prevents bone loss in ovariectomized mice. Cytotechnology.

[59] Sharma A.R., Lee Y.H., Bat-Ulzii A., Chatterjee S., Bhattacharya M., Chakraborty C., Lee S.S. (2023). Bioactivity, Molecular Mechanism, and Targeted Delivery of Flavonoids for Bone Loss. Nutrients.

[60] Ramesh P., Jagadeesan R., Sekaran S., Dhanasekaran A., Vimalraj S. (2021). Flavonoids: Classification, Function, and Molecular Mechanisms Involved in Bone Remodelling. Front. Endocrinol..

[61] Pan F.F., Shao J., Shi C.J., Li Z.P., Fu W.M., Zhang J.F. (2021). Apigenin promotes osteogenic differentiation of mesenchymal stem cells and accelerates bone fracture healing via activating Wnt/β-catenin signaling. Am. J. Physiol. Endocrinol. Metab..

[62] Quan H., Dai X., Liu M., Wu C., Wang D. (2019). Luteolin supports osteogenic differentiation of human periodontal ligament cells. BMC Oral Health.

[63] Bellavia D., Dimarco E., Costa V., Carina V., De Luca A., Raimondi L., Fini M., Gentile C., Caradonna F., Giavaresi G. (2021). Flavonoids in Bone Erosive Diseases: Perspectives in Osteoporosis Treatment. Trends Endocrinol. Metab..

[64] Torre E. (2017). Molecular signaling mechanisms behind polyphenol-induced bone anabolism. Phytochem. Rev..

[65] Tian C., Liu X., Chang Y., Wang R., Lv T., Cui C., Liu M. (2021). Investigation of the anti-inflammatory and antioxidant activities of luteolin, kaempferol, apigenin and quercetin. S. Afr. J. Bot..

[66] Bi J., Zhang C., Lu C., Mo C., Zeng J., Yao M., Jia B., Liu Z., Yuan P., Xu S. (2024). Age-related bone diseases: Role of inflammaging. J. Autoimmun..

[67] Luo J., Li L., Shi W., Xu K., Shen Y., Dai B. (2025). Oxidative stress and inflammation: Roles in osteoporosis. Front. Immunol..

[68] Foger-Samwald U., Kerschan-Schindl K., Butylina M., Pietschmann P. (2022). Age Related Osteoporosis: Targeting Cellular Senescence. Int. J. Mol. Sci..

[69] Marcucci G., Domazetovic V., Nediani C., Ruzzolini J., Favre C., Brandi M.L. (2023). Oxidative Stress and Natural Antioxidants in Osteoporosis: Novel Preventive and Therapeutic Approaches. Antioxidants.

[70] Cai Y., Sun H., Song X., Zhao J., Xu D., Liu M. (2023). The Wnt/β-catenin signaling pathway inhibits osteoporosis by regulating the expression of TERT: An in vivo and in vitro study. Aging.

[71] Brosche S., Strakova J., Jelinek N., Ochieng G., Otieno D., Saetang P., Boontongmai T., Bubphachat N., Thowsakul C., Sripumkhai P. (2025). Plastics Poison the Workplace II: Chemical Exposures to Plastic Waste and Recycling Workers in Kenya and Thailand.

[72] Colorado-Yohar S.M., Castillo-González A.C., Sánchez-Meca J., Rubio-Aparicio M., Sánchez-Rodríguez D., Salamanca-Fernández E., Ardanaz E., Amiano P., Fernández M.F., Mendiola J. (2021). Concentrations of bisphenol-A in adults from the general population: A systematic review and meta-analysis. Sci. Total Environ..

[73] Karinchai J., Budluang P., Temviriyanukul P., Ting P., Nuchuchua O., Wongnoppavich A., Imsumran A., Pitchakarn P. (2021). Bioassay-guided study of the anti-inflammatory effect of *Anoectochilus burmannicus* ethanolic extract in RAW 264.7 cells. J. Ethnopharmacol..

[74] Budluang P., Pitchakarn P., Ting P., Temviriyanukul P., Wongnoppawich A., Imsumran A. (2017). Anti-inflammatory and anti-insulin resistance activities of aqueous extract from *Anoectochilus burmannicus*. Food Sci. Nutr..

[75] Kant V., Mehta M., Varshneya C. (2012). Antioxidant potential and total phenolic contents of seabuckthorn (*Hippophae rhamnoides*) pomace. Free Radic. Antioxid..

[76] Gulcin İ., Alwasel S.H. (2023). DPPH Radical Scavenging Assay. Processes.

[77] Rumpf J., Burger R., Schulze M. (2023). Statistical evaluation of DPPH, ABTS, FRAP, and Folin-Ciocalteu assays to assess the antioxidant capacity of lignins. Int. J. Biol. Macromol..

[78] Cao G., Prior R.L. (1998). Comparison of different analytical methods for assessing total antioxidant capacity of human serum. Clin. Chem..

[79] Kanta P., Ghosh T., Kaur A., Muthukumarappa T. (2021). An innovative and cost-effective way to estimate alkaline phosphatase activity in in vitro cellular model systems. Int. J. Biochem. Mol. Biol..

[80] Campbell P.A. (2014). Alkaline Phosphatase Staining. Bio-Protocol.

[81] Bernar A., Gebetsberger J.V., Bauer M., Streif W., Schirmer M. (2022). Optimization of the Alizarin Red S Assay by Enhancing Mineralization of Osteoblasts. Int. J. Mol. Sci..

[82] Arter C., Trask L., Ward S., Yeoh S., Bayliss R. (2022). Structural features of the protein kinase domain and targeted binding by small-molecule inhibitors. J. Biol. Chem..

[83] Zhao W., Peng X., Yang F., Zhang Y., Wei Y., Huang J., Teng Y., Wan B., Zeng G., Zong S. (2026). Bisphenol A exacerbates osteoblast ferroptosis via the p53/SLC7A11 axis: A novel mechanistic insight into environmental osteoporosis pathogenesis. Ecotoxicol. Environ. Saf..

[84] Garcia-Recio E., Gonzalez-Acedo A., Manzano-Moreno F.J., De Luna-Bertos E., Ruiz C. (2024). Gene Expression Modulation of Markers Involved in Bone Formation and Resorption by Bisphenol A, Bisphenol F, Bisphenol S, and Bisphenol AF. Genes.

[85] Varma S., Molangiri A., Mudavath S., Ananthan R., Rajanna A., Duttaroy A.K., Basak S. (2024). Exposure to BPA and BPS during pregnancy disrupts the bone mineralization in the offspring. Food Chem. Toxicol..

[86] Martinand-Mari C., Debiais-Thibaud M., Potier E., Gasset E., Dutto G., Leurs N., Lallement S., Farcy E. (2024). Estradiol-17beta and bisphenol A affect growth and mineralization in early life stages of seabass. Comp. Biochem. Physiol. C Toxicol. Pharmacol..

[87] Fan J., Zhang D., Jiang Y., Yu L., Han B., Qian Z. (2023). The effects of PPARgamma inhibitor on bones and bone marrow fat in aged glucocorticoid-treated female rats. Exp. Gerontol..

[88] Nunes H.C., Tavares S.C., Garcia H.V., Cucielo M.S., Dos Santos S.A.A., Aal M.C.E., de Golim M.A., Justulin L.A., Ribeiro A.O., Deffune E. (2022). Bisphenol A and 2,3,7,8-tetrachlorodibenzo-p-dioxin at non-cytotoxic doses alter the differentiation potential and cell function of rat adipose-stem cells. Environ. Toxicol..

[89] Zhang Y., Yan M., Kuang S., Lou Y., Wu S., Li Y., Wang Z., Mao H. (2022). Bisphenol A induces apoptosis and autophagy in murine osteocytes MLO-Y4: Involvement of ROS-mediated mTOR/ULK1 pathway. Ecotoxicol. Env. Saf..

[90] Zhang Y., Yan M., Shan W., Zhang T., Shen Y., Zhu R., Fang J., Mao H. (2022). Bisphenol A induces pyroptotic cell death via ROS/NLRP3/Caspase-1 pathway in osteocytes MLO-Y4. Food Chem. Toxicol..

[91] Kim H.M., Lee S.M., Choi J., Soung N.K., Heo J.D. (2021). Effects of Bisphenol A and Its Alternatives, Bisphenol F and Tetramethyl Bisphenol F on Osteoclast Differentiation. Molecules.

[92] Thent Z.C., Froemming G.R.A., Ismail A.B.M., Fuad S., Muid S. (2018). Employing different types of phytoestrogens improve bone mineralization in bisphenol A stimulated osteoblast. Life Sci..

[93] Das S., Mukherjee U., Biswas S., Banerjee S., Karmakar S., Maitra S. (2024). Unravelling bisphenol A-induced hepatotoxicity: Insights into oxidative stress, inflammation, and energy dysregulation. Environ. Pollut..

[94] Shen Y., Li X., Wang H., Wang Y., Tao L., Wang P., Zhang H. (2023). Bisphenol A induced neuronal apoptosis and enhanced autophagy in vitro through Nrf2/HO-1 and Akt/mTOR pathways. Toxicology.

[95] Park S.J., Jang J.W., Moon E.Y. (2023). Bisphenol A-induced autophagy ameliorates human B cell death through Nrf2-mediated regulation of Atg7 and Beclin1 expression by Syk activation. Ecotoxicol. Environ. Saf..

[96] Jin Y., Yu C. (2025). Perinatal Exposure to Bisphenol A Induces Depressive-Like Behaviors, ERbeta Downregulation, and Dendritic Spine Loss in the Medial Amygdala. J. Appl. Toxicol..

[97] Tipbunjong C., Thitiphatphuvanon T., Pholpramool C., Surinlert P. (2024). Bisphenol-A Abrogates Proliferation and Differentiation of C2C12 Mouse Myoblasts via Downregulation of Phospho-P65 NF-kappaB Signaling Pathway. J. Toxicol..

[98] Mariem M., Slimen S., Stefania S., Gregorio P., Mourad J., Stefano D., Hichem S. (2025). Myrtus communis essential oil mitigates bisphenol A-induced reproductive and lipidomic alterations in a male rat model. Physiol. Rep..

[99] Giron-Perez M.I., Ventura-Ramon G.H., Covantes-Rosales C.E., Benitez-Trinidad A.B., Razura-Carmona F.F., Marmolejo-Murillo L.G., Perez-Arenivas C.E., Morales-Montor J., Diaz-Resendiz K.J.G. (2025). Effect of bisphenol-A and bisphenol-S on functional parameters of human leukocytes. Immunopharmacol. Immunotoxicol..

