# Supplementary material for: Wolffia globosa Ethanolic Extract Protects Against Bisphenol A-Induced Osteoblast Dysfunction via Antioxidant Defense, Apoptosis Inhibition, and β-Catenin Modulation
Source: Int J Mol Sci. 2026 Jun 13;27(12):5352. doi: 10.3390/ijms27125352 (PMC13299328; doi:10.3390/ijms27125352)
Supplement: Supplementary file 1 [file ijms-27-05352-s001.zip › ijms-4254252-supplementary.pdf]

Table S1 BPA Concentrations Employed in Cell Culture and *In Vivo* Studies

Study Model	Exposure Dose	Duration	Key Effects	Reference
Bone marrow–derived mesenchymal stem cells (BMSCs), MC3T3-E1 cells	0–500 µM (in vitro); 10 µg/kg (in vivo)	12-96 h; 21 days	BPA impaired osteoblast proliferation and differentiation, disrupted mineralization, and induced ferroptosis via p53/SLC7A11 signaling	[1]
Human osteoblast hFOB1.19 cells; zebrafish larvae	0-500 µM	96 h	Reduced cell viability and osteogenic markers (ALP, RUNX2); increased ROS and apoptosis (Caspase-3/9); suppression of osteogenic activity	[2]
Primary human osteoblasts (healthy donors)	10^{-5}–10^{-7} M	24 h.	Downregulation of RANKL, OPG, TGF-β1, and VEGF expression, indicating impaired bone remodeling signaling	[3]
SaOS-2 osteoblast-like cells; Wistar rats (offspring)	10 µM (in vitro); 0.4 µg/kg (in vivo)	72 h; 30–90 days	In vitro: cell viability and mineralization were decreased, ALP activity, altered BMP/TGF-β signaling were increased. In vivo: altered bone mineral content (BMC), bone mineral density (BMD), and plasma bone markers	[4]
European seabass (*Dicentrarchus labrax*) larvae	160 µg/L	7 days	Upregulation of osteoblast markers (sp7, bgp1b) and suppression of chondrocyte and osteoclast markers, indicating disrupted skeletal development	[5]
SaOS-2 osteoblast-like cells (in vitro); Wistar rats, pregnancy exposure (in vivo)	10 µM (in vitro); 0.4 µg/kg bw/day (oral gavage)	72 h; 30–90 day	BPA/BPS reduced osteoblast viability and mineralization, altered BMP/TGF-β signaling, and disrupted bone mineral content and density in offspring	[6]
Human MG-63 osteoblast-like osteosarcoma cells	0-10 µM)	7 days	BPA inhibited osteogenic differentiation via activation of RORα signaling	[7]

Study Model	Exposure Dose	duration	Key Effects	Reference
Rat mesenchymal stem cells	1-10 μM	7-14 days	High-dose BPA reduced viability, increased apoptosis and DNA damage, and altered differentiation potential.	[8]
Human osteoblast hFOB1.19 cells	10^{-5}–10^{-7} M	24 h; 21 days	BPA inhibited osteoblast growth, ALP synthesis, and mineralization via gene expression changes.	[9]
MLO-Y4 osteocytes	50-200 μM	24 h	BPA induced ROS-mediated autophagy and apoptosis through mTOR/ULK1 signaling.	[10]
MLO-Y4 osteocytes	200 μM	24 h	Induction of pyroptotic cell death via ROS/NLRP3/Caspase-1 pathway	[11]
RAW 264.7 osteoclast precursors	0-50 μM	48 -72 h	Modulation of RANKL-induced osteoclast differentiation via MAPK (JNK/p38) signaling	[12]
Human fetal osteoblasts (hFOB)	12.5 μg/mL	48 h	Reduced OPG production shifts balance toward osteoclast activation and bone resorption.	[13]
Male Swiss albino mice	0.5-50 mg/kg/day	30 days	Hepatotoxicity characterized by oxidative stress, NF-κB/NLRP3 activation, and metabolic dysregulation	[14]
SH-SY5Y neuroblastoma; PC12 cells	0-500 μM	6-24 h	BPA-induced neurotoxicity via ROS generation, mitochondrial dysfunction, apoptosis, and autophagy (Nrf2/HO-1, Akt/mTOR pathways).	[15]

Study Model	Exposure Dose	duration	Key Effects	Reference
Human B lymphoblast cells	100 μM	0-36 h	BPA-induced autophagy promoting cell survival through Syk-Nrf2-Atg7/Beclin1 signaling	[16]
C57BL/6J mice (perinatal exposure)	50 μg/kg/day	8 weeks	Anxiety- and depression-like behaviors associated with Erβ downregulation and synaptic alterations	[17]
C2C12 myoblasts	25-50 μM	72 h	Suppressed myoblast proliferation and differentiation via NF-κB signaling inhibition	[18]
Male Wistar rats	100 μg/kg/day	30 days	Reproductive toxicity marked by oxidative stress, hormonal disruption, and altered lipid composition	[19]
Human leukocytes	1-500 ng/mL	24 h	Mitochondrial dysfunction, increased ROS, senescence, and calcium dysregulation, indicating immunotoxicity	[20]

References

1. Zhao, W.; Peng, X.; Yang, F.; Zhang, Y.; Wei, Y.; Huang, J.; Teng, Y.; Wan, B.; Zeng, G.; Zong, S. Bisphenol A exacerbates osteoblast ferroptosis via the p53/SLC7A11 axis: A novel mechanistic insight into environmental osteoporosis pathogenesis. *Ecotoxicology and Environmental Safety* **2026**, *309*, 119560, doi:https://doi.org/10.1016/j.ecoenv.2025.119560.
2. Shi, X.; Wu, K.; Liu, C.; Cao, K.; Zhang, Q.; Wu, W.; Luo, C.; Huang, W. Preliminary investigation into the impact of BPA on osteoblast activity and bone development: In vitro and in vivo models. *Environ Pollut* **2024**, *347*, 123731, doi:10.1016/j.envpol.2024.123731.
3. Garcia-Recio, E.; Gonzalez-Acedo, A.; Manzano-Moreno, F.J.; De Luna-Bertos, E.; Ruiz, C. Gene Expression Modulation of Markers Involved in Bone Formation and Resorption by Bisphenol A, Bisphenol F, Bisphenol S, and Bisphenol AF. *Genes (Basel)* **2024**, *15*, doi:10.3390/genes15111453.
4. Varma, S.; Molangiri, A.; Mudavath, S.; Ananthan, R.; Rajanna, A.; Duttaroy, A.K.; Basak, S. Exposure to BPA and BPS during pregnancy disrupts the bone mineralization in the offspring. *Food Chem Toxicol* **2024**, *189*, 114772, doi:10.1016/j.fct.2024.114772.
5. Martinand-Mari, C.; Debiais-Thibaud, M.; Potier, E.; Gasset, E.; Dutto, G.; Leurs, N.; Lallement, S.; Farcy, E. Estradiol-17beta and bisphenol A affect growth and mineralization in early life stages of seabass. *Comp Biochem Physiol C Toxicol Pharmacol* **2024**, *281*, 109921, doi:10.1016/j.cbpc.2024.109921.
6. Fan, J.; Zhang, D.; Jiang, Y.; Yu, L.; Han, B.; Qian, Z. The effects of PPARgamma inhibitor on bones and bone marrow fat in aged glucocorticoid-treated female rats. *Exp Gerontol* **2023**, *181*, 112281, doi:10.1016/j.exger.2023.112281.
7. Maduranga Karunarathne, W.A.H.; Choi, Y.H.; Park, S.R.; Lee, C.M.; Kim, G.Y. Bisphenol A inhibits osteogenic activity and causes bone resorption via the activation of retinoic acid-related orphan receptor alpha. *J Hazard Mater* **2022**, *438*, 129458, doi:10.1016/j.jhazmat.2022.129458.

8. Nunes, H.C.; Tavares, S.C.; Garcia, H.V.; Cucielo, M.S.; Dos Santos, S.A.A.; Aal, M.C.E.; de Golim, M.A.; Justulin, L.A., Jr.; Ribeiro, A.O.; Deffune, E.; et al. Bisphenol A and 2,3,7,8-tetrachlorodibenzo-p-dioxin at non-cytotoxic doses alter the differentiation potential and cell function of rat adipose-stem cells. *Environ Toxicol* **2022**, *37*, 2314-2323, doi:10.1002/tox.23598.
9. Garcia-Recio, E.; Costela-Ruiz, V.J.; Melguizo-Rodriguez, L.; Ramos-Torrecillas, J.; Garcia-Martinez, O.; Ruiz, C.; de Luna-Bertos, E. Repercussions of Bisphenol A on the Physiology of Human Osteoblasts. *Int J Mol Sci* **2022**, *23*, doi:10.3390/ijms23105349.
10. Zhang, Y.; Yan, M.; Kuang, S.; Lou, Y.; Wu, S.; Li, Y.; Wang, Z.; Mao, H. Bisphenol A induces apoptosis and autophagy in murine osteocytes MLO-Y4: Involvement of ROS-mediated mTOR/ULK1 pathway. *Ecotoxicol Environ Saf* **2022**, *230*, 113119, doi:10.1016/j.ecoenv.2021.113119.
11. Zhang, Y.; Yan, M.; Shan, W.; Zhang, T.; Shen, Y.; Zhu, R.; Fang, J.; Mao, H. Bisphenol A induces pyroptotic cell death via ROS/NLRP3/Caspase-1 pathway in osteocytes MLO-Y4. *Food Chem Toxicol* **2022**, *159*, 112772, doi:10.1016/j.fct.2021.112772.
12. Kim, H.M.; Lee, S.M.; Choi, J.; Soung, N.K.; Heo, J.D. Effects of Bisphenol A and Its Alternatives, Bisphenol F and Tetramethyl Bisphenol F on Osteoclast Differentiation. *Molecules* **2021**, *26*, doi:10.3390/molecules26206100.
13. Thent, Z.C.; Froemming, G.R.A.; Ismail, A.B.M.; Fuad, S.; Muid, S. Employing different types of phytoestrogens improve bone mineralization in bisphenol A stimulated osteoblast. *Life Sci* **2018**, *210*, 214-223, doi:10.1016/j.lfs.2018.08.057.
14. Das, S.; Mukherjee, U.; Biswas, S.; Banerjee, S.; Karmakar, S.; Maitra, S. Unravelling bisphenol A-induced hepatotoxicity: Insights into oxidative stress, inflammation, and energy dysregulation. *Environ Pollut* **2024**, *362*, 124922, doi:10.1016/j.envpol.2024.124922.
15. Shen, Y.; Li, X.; Wang, H.; Wang, Y.; Tao, L.; Wang, P.; Zhang, H. Bisphenol A induced neuronal apoptosis and enhanced autophagy in vitro through Nrf2/HO-1 and Akt/mTOR pathways. *Toxicology* **2023**, *500*, 153678, doi:10.1016/j.tox.2023.153678.
16. Park, S.J.; Jang, J.W.; Moon, E.Y. Bisphenol A-induced autophagy ameliorates human B cell death through Nrf2-mediated regulation of Atg7 and Beclin1 expression by Syk activation. *Ecotoxicol Environ Saf* **2023**, *260*, 115061, doi:10.1016/j.ecoenv.2023.115061.
17. Jin, Y.; Yu, C. Perinatal Exposure to Bisphenol A Induces Depressive-Like Behaviors, ERbeta Downregulation, and Dendritic Spine Loss in the Medial Amygdala. *J Appl Toxicol* **2025**, *45*, 2344-2352, doi:10.1002/jat.4845.
18. Tipbunjong, C.; Thitiphatphuvanon, T.; Pholpramool, C.; Surinlert, P. Bisphenol-A Abrogates Proliferation and Differentiation of C2C12 Mouse Myoblasts via Downregulation of Phospho-P65 NF-kappaB Signaling Pathway. *J Toxicol* **2024**, *2024*, 3840950, doi:10.1155/2024/3840950.
19. Mariem, M.; Slimen, S.; Stefania, S.; Gregorio, P.; Mourad, J.; Stefano, D.; Hichem, S. Myrtus communis essential oil mitigates bisphenol A-induced reproductive and lipidomic alterations in a male rat model. *Physiol Rep* **2025**, *13*, e70628, doi:10.14814/phy2.70628.
20. Giron-Perez, M.I.; Ventura-Ramon, G.H.; Covantes-Rosales, C.E.; Benitez-Trinidad, A.B.; Razura-Carmona, F.F.; Marmolejo-Murillo, L.G.; Perez-Arenivas, C.E.; Morales-Montor, J.; Diaz-Resendiz, K.J.G. Effect of bisphenol-A and bisphenol-S on functional parameters of human leukocytes. *Immunopharmacol Immunotoxicol* **2025**, *47*, 621-630, doi:10.1080/08923973.2025.2542133.
